# A myriad of roles of miR-25 in health and disease

**DOI:** 10.18632/oncotarget.24662

**Published:** 2018-04-20

**Authors:** Márta Sárközy, Zsuzsanna Kahán, Tamás Csont

**Affiliations:** ^1^ Department of Biochemistry, Faculty of Medicine, University of Szeged, H-6720 Szeged, Hungary; ^2^ Department of Oncotherapy, Faculty of Medicine, University of Szeged, H-6720 Szeged, Hungary

**Keywords:** cardiovascular diseases, oncology, p57, SERCA2a, TRAIL

## Abstract

Small non-coding RNAs including microRNAs (miRNAs) have been recently recognized as important regulators of gene expression. MicroRNAs play myriads of roles in physiological processes as well as in the pathogenesis of a number of diseases by translational repression or mRNA destabilization of numerous target genes. The miR-106b-25 cluster is highly conserved in vertebrates and consists of three members including miR-106b, miR-93 and miR-25. MiR-106b and miR-93 share the same seed sequences; however, miR-25 has only a similar seed sequence resulting in different predicted target mRNAs. In this review, we specifically focus on the role of miR-25 in healthy and diseased conditions. Many of miR-25 target mRNAs are involved in biological processes such as cell proliferation, differentiation, and migration, apoptosis, oxidative stress, inflammation, calcium handling, etc. Therefore, it is no surprise that miR-25 has been reported as a key regulator of common cancerous and non-cancerous diseases. MiR-25 plays an important role in the pathogenesis of acute myocardial infarction, left ventricular hypertrophy, heart failure, diabetes mellitus, diabetic nephropathy, tubulointerstitial nephropathy, asthma bronchiale, cerebral ischemia/reperfusion injury, neurodegenerative diseases, schizophrenia, multiple sclerosis, etc. MiR-25 is also a well-described oncogenic miRNA playing a crucial role in the development of many tumor types including brain tumors, lung, breast, ovarian, prostate, thyroid, oesophageal, gastric, colorectal, hepatocellular cancers, etc. In this review, our aim is to discuss the translational therapeutic role of miR-25 in common diseased conditions based on relevant basic research and clinical studies.

## INTRODUCTION

### MicroRNAs and their biogenesis

Although almost 85% of the human genome is known to be transcribed [1, 2], only 2% of the genome codes for proteins. The vast majority of the genome yields non-coding RNAs including long and small non-coding RNAs [3, 4]. MicroRNAs (miRNAs, miRs) are a dominating class of small noncoding RNAs in most somatic tissues [3, 4]. They are approximately 21-25 nucleotide in length and their major function is to mediate post-transcriptional gene silencing [3, 4].

**Figure 1:**
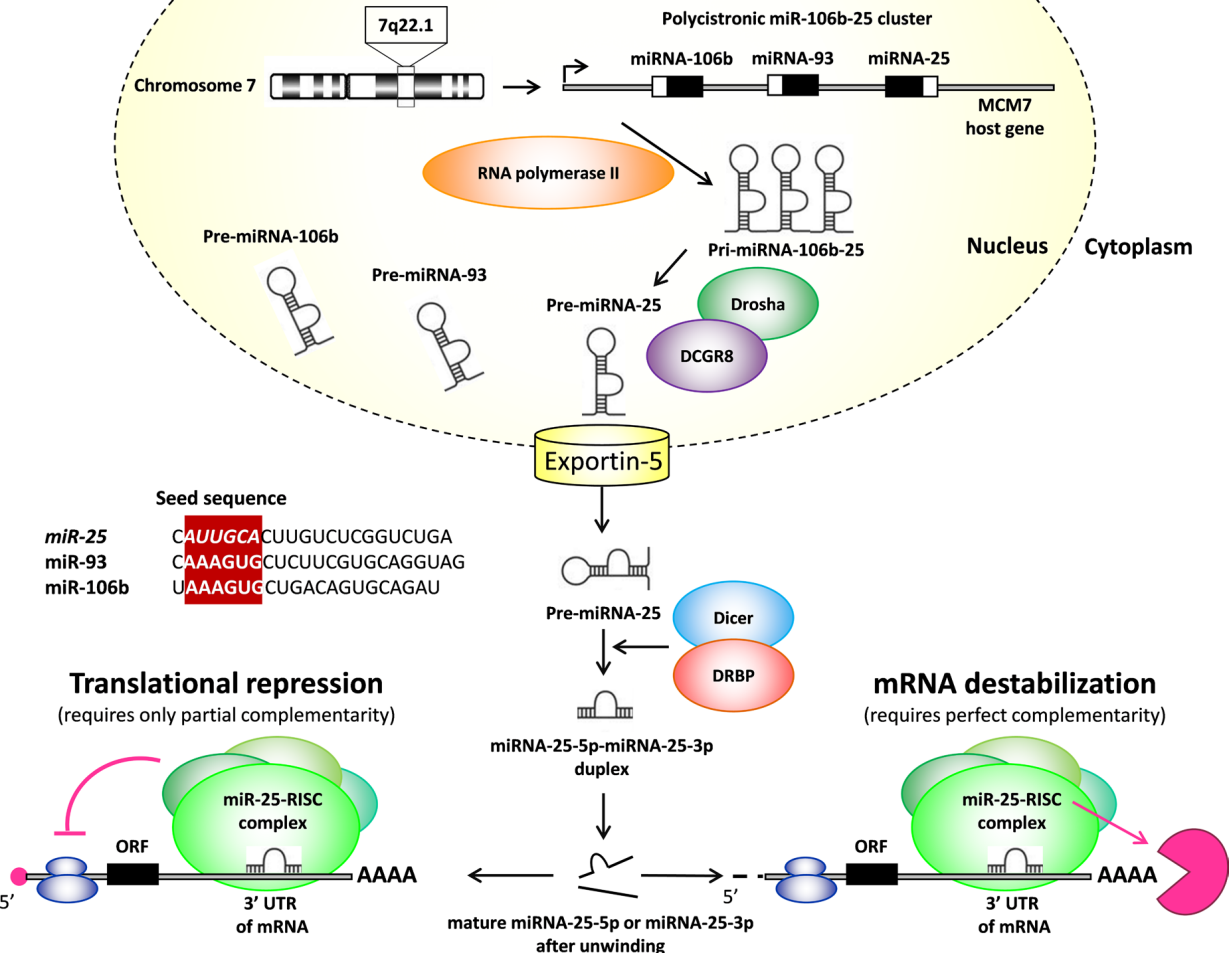
Maturation of miR-25 UTR: untranslated region, ORF: open reading frame.

A detailed description of the miRNA biogenesis is beyond the scope of this review. Therefore, here we just briefly mention some important aspects and otherwise refer to excellent reviews for more details (Figure 1) [4–6]. MicroRNA genes are processed either from introns of protein-coding genes or dedicated miRNA gene loci [7, 8]. An individual primary transcript can either produce a single miRNA or generate a miRNA cluster containing two or more miRNAs [7]. According to our current knowledge, microRNA genes are transcribed by RNA polymerase II as primary microRNAs (pri-microRNAs) (Figure 1). A typical pri-miRNA consists of a stem of 33-35 bp, a terminal loop and single-stranded RNA segments at both the 5’ and 3’ sites [5]. Pri-miRNAs are cleaved by the Microprocessor complex containing the RNase III enzyme Drosha and its essential cofactor DGCR8 and form precursor microRNAs (pre-miRNAs) in the nucleus (Figure 1) [5]. Drosha has tandem RNase III domains which dimerize to form one processing center [5]. The first RNase III domain cuts the 3’ strand of the stem of pri-miRNA and the second RNase III domain cuts the 5’ strand. The produced pre-microRNAs are double-stranded and approximately 70 nucleotide in length, and have a terminal loop [4–6] (Figure 1). These pre-miRNAs are subsequently transported to the cytoplasm by exportin-5 where their terminal loops are cleaved by another RNase III enzyme (DICER) to form a small RNA (miR-miR^*^) duplex [4–6] (Figure 1). DICER cleavage sites are located at a 21-25 nucleotides distance from the 3’ end and 22 nucleotides away from the 5’ end. This small RNA duplex is loaded onto an Argonaute (AGO) protein to form an effector complex called RNA-induced silencing complex (RISC) [4–6] (Figure 1). Then the two strands of the small RNA duplex are unwinded, and generally, only one strand will be the single-stranded mature microRNA (miR), and the other strand will be degraded (miR^*^ or passenger strand) [4–6] (Figure 1). The guide strand is determined during the AGO loading process and generally becomes later the mature miR [5]. The strand at 5’ side is typically selected as the guide strand (miR-5p) [5]. Strand selection is not completely strict. Therefore, the not-favored and less abundant passenger strand at the 3’ side could also be selected and act as mature miRNA (miR-3p). Alternative strand selection (5p to 3p arm switching) could be tissue-specific [5]. Mature miRNAs can either inhibit the translation of target mRNAs or promote their destabilization and degradation through imperfect sequence-specific binding to the 3’ untranslated region (3’- UTR) of target mRNAs [5, 6] (Figure 1). Individual miRNAs may simultaneously target multiple mRNAs. Moreover, the expression of individual mRNAs can be regulated by multiple miRNAs. Therefore, miRNAs may act as fine tuners or as on/off switches of gene expression [9, 10] (Figure 1).

Classification rules of miRNAs have not yet been unified. It is generally considered that miRNAs with identical seed sequences at nucleotides 2-8 of the mature miRNA belong to the same miRNA (“seed”) family [5]. The occurrence of miRNAs belonging to distinct “seed” families within the same cluster is also commonly observed [5]. The seed sequence corresponds to the 3’ UTR of the target mRNAs and determines the possible mRNA targets of the miRNA [5, 7].

Since the original discovery of the first miRNA, lin-4 in 1993 [11] and the second miRNA, let-7 in *Caenorhabditis elegans* [11–14], the exponentially increasing number of studies indicate that the functions of miRNAs are not limited to the regulation of developmental events. MicroRNAs also regulate many other aspects of biological processes in animals and plants including oxidative stress, cell death, cell proliferation, etc. in many tissue types [15–18]. Therefore, dysregulation of miRNAs in pathological conditions may alter gene networks. Consequently, miRNA replacement or anti-sense inhibition therapy offers a new approach to treat diseases by modulating gene pathways rather than single molecular targets [19].

### MicroRNA-25

The miR-106b/25 cluster is highly conserved in vertebrates [20]. The three members of the cluster, miR-106b, miR-93, and miR-25 are located in a 515 bp region on chromosome 7q22 in intron 13 of the host gene minichromosome maintenance protein 7 (MCM7). These three miRNAs are co-transcribed with their host gene [6, 20, 21]. The host gene MCM7 is a component of the highly conserved MCM2-7 complex (MCM complex) [6, 20, 21]. The MCM complex is a member of DNA helicases which are essential in the initiation of DNA replication in eukaryotic cells [8]. MiR-106b and miR-93 share the same seed sequences; however, miR-25 has only a similar seed sequence resulting in different predicted target mRNAs [20]. In this review, we focus on the role of miR-25 in healthy and diseased conditions. The mature miR-25 (miR-25-3p) consists of 22 nucleotides (CAUU GCAC UUGU CUCG GUCU GA) (ww and www.miRbase.org) and has 1163 predicted target mRNA transcripts with conserved sites (TargetScanHuman version 7.1). Mature miR-25 belongs to the evolutionary broadly conserved miR-25-3p/32-5p/92-3p/363-3p/367-3p seed family and has the same predicted mRNA targets as the other miRNA members of this seed family (TargetScanHuman version 7.1). The mature miR-25^*^ (miR-25-5p) consists of 22 nucleotides (AGGC GGAG ACAC GGGC AAUU GC) (https://targetexplorer.ingenuity.com/index.htm and www.miRbase.org) and has 1868 predicted mRNA transcripts; however, these predicted targets are primarily false positives (TargetScanHuman version 7.1).

### The role of miR-25 in health

**Figure 2:**
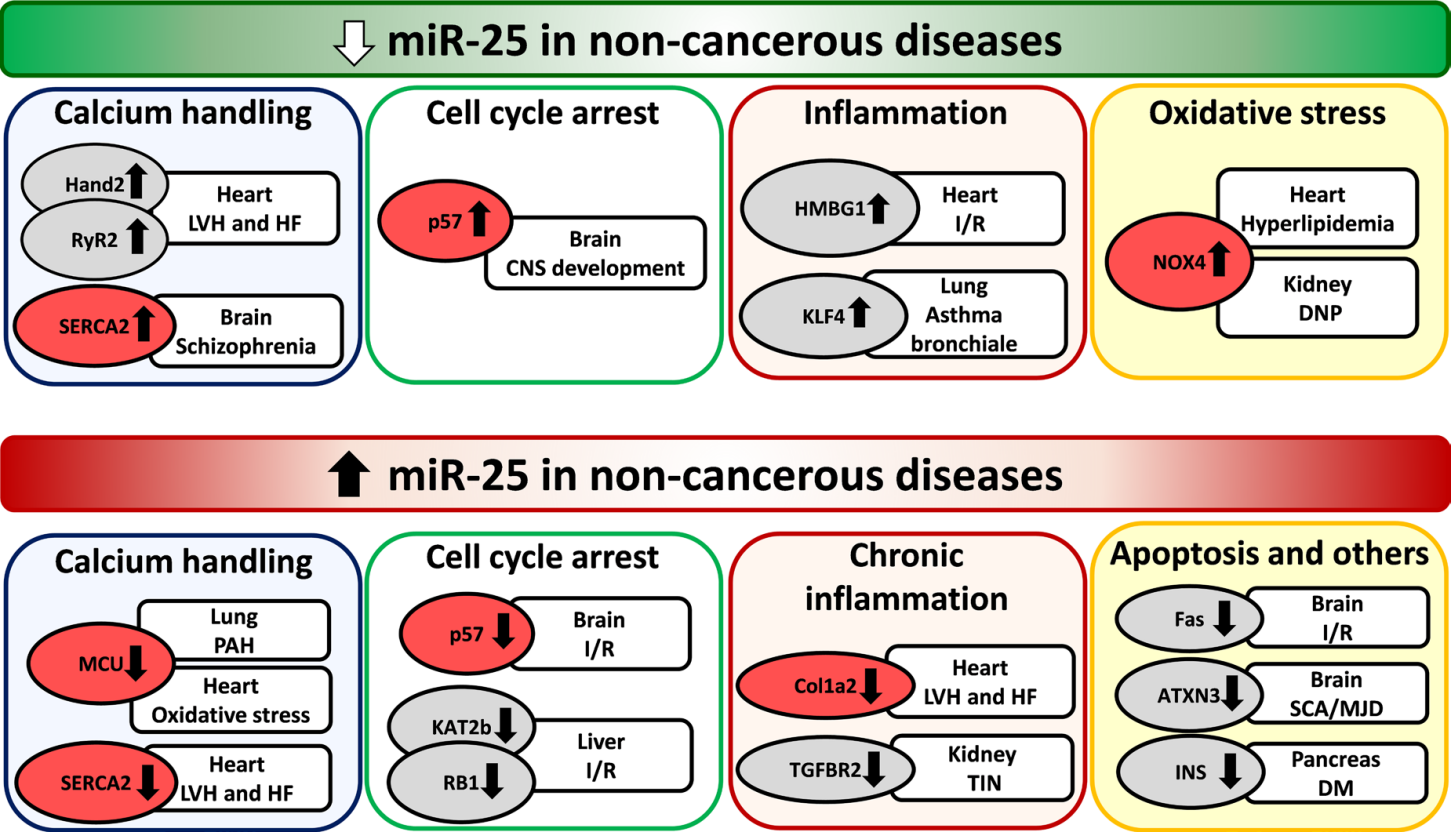
Repression and overexpression of miR-25 in non-cancerous diseases CNS: central nervous system, DM: diabetes mellitus, DNP: diabetic nephropathy, LVH: left ventricular hypertrophy, HF: heart failure, I/R: ischemia/reperfusion, PAH: pulmonary arterial hypertension, SCA/MJD: Spinocerebellar ataxia type 3/Machado-Joseph Disease, TIN: tubulointerstitial nephropathy. Gene symbols in bubbles are targets of miR-25 in multiple organs/diseases. Genes in red bubbles are targets of miR-25 in multiple diseases.

It is very difficult to find literature about the function of miR-25 in healthy conditions in the PubMed database. Surprisingly, there is only a few relevant research paper in the PubMed database using the following keywords: miR-25, health; miR-25, physiological conditions; miR-25, normal conditions; miR-25, development [22–24]. Therefore, we can only deduce the possible roles of miR-25 in healthy conditions from research papers using miR-25 overexpressing or loss of function cells or animals. Under physiological conditions, mature miR-25 seems to play a crucial role in the regulation of developmental events [22, 23]. According to the AMIGO 2 gene ontology database, miR-25 is a negative regulator of cardiac muscle growth and cardiac cell development (GO:0055022). Therefore, it is no surprise that miR-25 also play an important role in the development of cardiac hypertrophy and heart failure under pathophysiological conditions (Figure 2). Under normal conditions, many of miR-25 target mRNAs are involved in biological processes such as response to DNA damage, cell cycle regulation, cell proliferation, migration, and differentiation. In addition, many of miR-25 target molecules can be found among extracellular matrix components and membrane receptors. Under pathophysiological conditions, miR-25 is also a well-described oncogenic miRNA. It plays a crucial role in the development and spread of many tumor types including brain tumors, lung, breast, ovarian, prostate, thyroid, esophageal, gastric, colorectal, hepatocellular cancers, etc. Other groups of miR-25 target molecules are important regulators of apoptosis, autophagy, oxidative stress, inflammation, calcium handling, etc. These mechanisms could be key factors in the pathogenesis of acute myocardial infarction, heart failure, diabetes mellitus, diabetic nephropathy, tubulointerstitial nephropathy, asthma bronchiale, cerebral ischemia/reperfusion injury, neurodegenerative diseases, schizophrenia, multiple sclerosis, etc. In this review, we aim to provide an in-depth discussion of the translational therapeutic role of miR-25 in diseased conditions based on relevant basic research articles and clinical studies.

### The role of miR-25 in diseased conditions

#### Non-cancerous diseases

##### Cardiovascular diseases

#### Acute coronary syndrome

The acute coronary syndrome (ACS) is the leading cause of morbidity and mortality in the industrialized countries. ACS represents a spectrum of coronary artery diseases including unstable angina (UA), non-ST-segment elevation myocardial infarction (NSTEMI) and ST-segment elevation myocardial infarction (STEMI) [25]. Acute myocardial infarction in patients is related to rupture-prone or vulnerable atherosclerotic plaques [26]. A cohort study recruiting 13 patients with non-cardiac chest pain and 13 patients with UA and angiographically-proven coronary artery disease reported that members of the miR-106b/25 cluster were significantly elevated in plasma samples of UA patients [26] (Table 1). Another cohort study recruiting 13 NSTEMI and 13 STEMI patients showed that the expression of miR-25-3p was significantly increased in the blood of STEMI patients as compared to NSTEMI patients (Table 1). According to the aforementioned clinical studies, circulating miR-25 seems to be a potential biomarker in ACS. However, both studies have many limitations including the small sample size, non-diverse genetic, social, and treatment characteristics of ACS patients. Moreover, investigation of the time dependency of miR-25 upregulation in ACS patients would be more informative. In contrast to these clinical observations, the expression of miR-25 failed to show any change in response to 30-min ischemia and 120-min reperfusion in isolated perfused hearts of male Wistar rats in our previous preclinical study [27]. Nevertheless, the event of 30-min coronary occlusion followed by 120-min reperfusion was considered an early phase of cell injury due to necrotic and/or apoptotic processes [27]. Moreover, since only a single time point was used upon *ex vivo* reperfusion for heart sample harvesting, the time course of miRNA expression changes remained unknown in heart tissue and blood [27].

**Table 1:** The role of miR-25 in cardiovascular and renal diseases

	Disease	Species and tissue or cell type	Alteration of miR-25 expression	Method for miR-25 detection	Target gene	Biological function	Method for target validation	Sample size in clinical studies	Ref.
1	ACS - NSTEMI vs. STEMI	Human BP (American)	up-regulation	Qiagen miRNeasy kit	N/A	N/A	N/A	9 STEMI vs. 4 NSTEMI	[25]
2	ACS - UA	Human BP (Chinese)	up-regulation (miR-106b-25 cluster)	Taqman low density miRNA array	N/A	N/A	N/A	13 UA vs. 13 CONT	[26]
3	H/R	H9c2 cells (24 h I/1 h R)	*down-regulation*	qRT-PCR	HMGB1	inflammation	**DLRA**, miR-25 TF	N/A	[33]
4	LVH and HF	heart of TAC mice (4,8,10 wk) and human HF	*down-regulation*	Affymetrix GeneChip Mouse Gene St 1.0 Array, Northern blot, qRT-PCR	Hand2	hypertrophy and fibrosis	**LRA**, MHC-Hand2 TG mice, miR-25 TF, antagomiR-25	N/A	[30]
5	LVH and HF	heart of SMAD3−/− TAC mice (10-20 days), cardiac FB	up-regulation	miRNA array, qRT-PCR	Col1a2	fibrosis	miR-25 TF	N/A	[32]
6	LVH and HF	heart of TAC mice (22 wk), and human HF, HEK293 cells and RCm	up-regulation	miRNA array, qRT-PCR	SERCA2a	calcium handling	**LRA**, miR-25 OE mice, SERCA2a KO mice, antagomiR-25 assay	N/A	[34]
7	LVH and HF	uninephrectomized and salt-fed rat myocardium (20 wk) and plasma	up-regulation (myocardium)*down-regulation* (plasma)	qRT-PCR	SERCA2a	calcium handling	Western blot	N/A	[36]
8	Hyper-cholesterolemia	rat myocardium (12 wk) and RCm	*down-regulation*	Agilent's microRNA complate labeling and hyb system, qRT-PCR	NOX4	oxidative stress	**LRA**	N/A	[39]
9	Paroxysmal atrial fibrillation	human RA (German), heart of miR-25/106b−/− mice, H9c2 cells	*down-regulation*	qRT-PCR	RyR2	calcium handling	**LRA**	8 pAF vs. 10 CONT	[37]
10	Atherosclerosis and vascular remodelling	human aortic VSMCs	*down-regulation of miR-25-5p*	Affymetrix GeneChip microRNA Array and qRT-PCR	N/A	N/A	N/A	N/A	[41]
11	Intracranial aneurysm	Human BP (Chinese)	up-regulation	Agilent's microRNA complate labeling and hyb system, qRT-PCR	N/A	N/A	N/A	40 IA vs. 20 CONT	[42]
12	Pulmonary arterial hypertension	human PASMCs, rat	up-regulation	Affymetrix GeneChip miRNA 4.0 Array, Northern blot, qRT-PCR	MCU	apoptosis	**LRA**, MCU OE, antagomiR-25 in rats	6 PAH vs. 3 CONT	[43]
13	T1DM	male Wistar rats (3 wk HFD+STZ), INS-1 cells	up-regulation	Exiqon miRCURY LNA array, qRT-PCR	insulin	glucose homeostasis	**LRA**, miR-25 TF	N/A	[45]
14	T1DM	human BS (European multicenter and Danish cohort)	up-regulation	Illumina Solexa Sequencing	N/A	N/A	N/A	275 European T1DM, 129 Danish T1DM and 151 CONT	[46]
15	TIN	HK-2 cell line (human)	*down-regulation (miR-106b-25 cluster)*	Exiqon miRCURY LNA array, qRT-PCR	TGFBR2	fibrosis	WB, miR-106b TF	N/A	[20]
16	DNP	mesangial cells from male SD rats (12 wk, STZ)	*down-regulation*	qRT-PCR	NOX4	oxidative stress	**LRA**	N/A	[48]
17	IgA NP	human urine (Chinese)	up-regulation	Agilent human miRNA microarray V19.0, qRT-PCR	N/A	N/A	N/A	93 IgA NP vs. 82 CONT	[49]

Abbreviations: ACS: acute coronary syndrome, BP: blood plasma, BS: blood serum, CONT: control, DLRA: dual luciferase reporter assay, DNP: diabetic nephropathy, FB: fibroblast, IF: immunofluorescence, HF: heart failure, HFD: high fat diet, H/R: hypoxia/reoxygenation, IA: intracranial aneurysm, IgA NP: IgA nephropathy, IH: immunohistochemistry, LRA: luciferase reporter assay, LVH: left ventricular hypertrophy, KO: knock out, NSTEMI: non ST-elevation myocardial infarction, OE: overexpression, pAF: paroxysmal atrial fibrillation, PAH: pulmonary arterial hypertension, PASMCs: pulmonary artery smooth muscle cells, RA: right atrium, RCm: rat cardiomyocytes, SD: Sprague Dawley, STEMI: ST-elevation myocardial infarction, STZ: streptozotocin, T1DM: type 1 diabetes mellitus, TAC: transverse aortic constriction, TIN: tubulointerstitial nephropathy, TF: transfection, TG: transgene, UA: unstable angina, VSMC: vascular smooth muscle cell, WB: Western blot, wk: week. The abbreviations in bold are considered as gold standard methods for miRNA target validation.

#### Left ventricular hypertrophy and heart failure

Left ventricular hypertrophy (LVH) defined as increase in cardiomyocyte size secondary to i) increased mechanical load (e.g. hypertension, valvular disease, etc.), ii) decreased mechanical performance (e.g. ischemic heart disease, myocarditis, contractile protein mutations, etc.), iii) increased neurohumoral activity, and iv) hereditary cardiomyopathies with seemingly normal performance and load [28–30]. During the development of LVH, a fetal cardiac gene program is activated by a defined set of transcription factors as an initially adaptive response to stress [29, 30]. The abnormal expression of fetal proteins includes contractile elements (e.g., alpha-MHC, beta-MHC), ECM matrix components (e.g., collagens), calcium handling (e.g., SERCA2a) and mitochondrial proteins (e.g., oxidized elements of the electron transport chain), etc. These changes lead to a maladaptive response to stress resulting in the development of fibrosis, heart failure (HF) and fatal arrhythmias [29, 30]. From a clinical point of view, heart failure could be classified as heart failure with preserved ejection fraction (HFpEF, i.e., left vetricular ejection fraction (LVEF)>50%), heart failure with reduced ejection fraction (HFrEF, LVEF <40%) and most recently heart failure with mid-range ejection fraction (HFmrEF, LVEF 40–49%) [31].

An experimental study proved that transforming growth factor-β1 (TGF-β1) signaling leads to cardiac hypertrophy and fibrosis through a Mothers Against Decapentaplegic Homolog 3 (SMAD3)-dependent manner 20 days after transverse aortic constriction (TAC) in SMAD3^−/−^ and littermate control mice [32] (Table 1). This study also demonstrated that miR-25 transfection into isolated cardiac fibroblasts decreased collagen-1a2 expression [32] (Table 1). In another study, H9c2 cells subjected to hypoxia/reoxygenation showed decreased expression of miR-25 and increased level of its direct target high-mobility group box 1 (HMGB1). HMGB1 is known as a regulator of epithelial to mesenchymal transition and as an inflammatory as well as a pro-fibrotic factor [33] (Table 1). H9c2 cells showed increased levels of apoptosis (caspase-3) and fibrosis markers (collagen I, collagen III, fibronectin, TIMP1, and MM2) after hypoxia/reoxygenation [33] (Table 1). The overexpression of miR-25 resulted in inhibition of apoptosis and fibrosis through down-regulation of HMBG1 after hypoxia/reoxygenation in H9c2 cells [33] (Figure 2). These anti-apoptotic and anti-fibrotic effects of miR-25 could be further enhanced by a TGF-β1/SMAD3 inhibitor (SB431542) [33]. Therefore, miR-25 and HMGB1 seem to be early regulators of the TGF-β1/SMAD3 signaling pathway which is a key factor in the development of cardiac fibrosis and heart failure.

Dirkx et al. and Wahlquist et al. investigated the role of miR-25 in calcium handling and cardiac remodeling in TAC-induced LVH and HF models and also in human failing heart samples. However, their results are rather controversial [30, 34] (Table 1). Dirkx et al. found in a mouse model 20 days after TAC surgery that down-regulation of miR-25 and overactivation of the calcineurin/Nfat signaling resulted in overactivation of the Hand2 transcription factor and its target genes leading to cardiac fibrosis and heart failure [30] (Table 1) (Figure 2). In contrast, Wahlquist et al. reported that cardiac overexpression of miR-25 could lead to LVH and HF 5.5 months after TAC surgery in mice. Moreover, inhibition of miR-25 could ameliorate contractile dysfunction by improving sarcoendoplasmic reticulum calcium ATPase 2 (SERCA2a) activity and Ca^2+^ handling [34] (Table 1) (Figure 2). In response to these controversial results, Bush et al. wrote a commentary on the potential causes including differences in experiment durations and follow-up times after TAC surgery, as well as different antagomiR-25 chemistries and doses [35].

Interestingly, it was found in LVH and HF induced by uninephrectomy and high salt (10% w/w NaCl) diet for 20 weeks that cardiac miR-25 level was significantly up-regulated and its direct target SERCA2 level was significantly decreased [36] (Table 1) (Figure 2). In contrast, circulating miR-25 concentration was significantly decreased in LVH and HF rats as compared to controls [36] (Table 1).

These five articles showed that miR-25 expression is regulated in a time-dependent manner in cardiac hypertrophy, fibrosis, and heart failure. MiR-25 mimics could play a beneficial role in early phases of cardiac remodeling and heart failure. However, overexpression of miR-25 could be detrimental due to maladaptive effects in chronic heart failure. Since miRNA levels in cardiac tissue and circulation are not always concordant, caution must be exercised during the utilization of miRs as diagnostic or prognostic biomarkers.

#### Arrhythmias

It is also well-known that increased sarcoplasmic reticulum (SR) Ca^2+^-leak via ryanodine receptor type-2 (RyR2) contributes to the pathogenesis of atrial fibrillation (AF). Interestingly, the expression of miR-25 was decreased in atria of patients with paroxysmal atrial fibrillation (PAF) compared with patients with sinus rhythm [37] (Table 1). Moreover, this study showed that miR-106b-25^−/−^ mice expressed increased atrial RyR2 protein levels as well as SR Ca^2+^-leak, and were more prone to atrial ectopy than wild-type littermates [37] (Table 1) (Figure 2).

#### Hypercholesterolemia

Hypercholesterolemia is a well-known risk factor for cardiovascular diseases, and it leads to increased oxidative/nitrative stress in the myocardium [38]. Experimental data are very limited on the regulatory role of miRNAs in hypercholesterolemia-induced cardiac pathologies. We have previously shown that diet-induced hypercholesterolemia in male Wistar rats (2% cholesterol- and 0.25% sodium cholate-enriched diet for 12 weeks) leads to the down-regulation of miR-25 in the myocardium [39] (Table 1). Subsequently, the superoxide-generating NADPH oxidase 4 (NOX4) which is a direct target of miR-25, was up-regulated in the myocardium. In the same study, cardiac oxidative/nitrative stress was also increased leading to diastolic dysfunction in hypercholesterolemic rats [39] (Table 1) (Figure 2). Moreover, knock-down of miR-25 could significantly increase the oxidative stress and NOX4 protein levels in neonatal rat cardiomyocytes 24 hours after transfection of a miR-25 inhibitor proving the direct link between miR-25 and NOX4 expression [39] (Table 1) (Figure 2). However, in another study it was reported that oxidative stress induced by 2 or 3 hours of H_2_O_2_-incubation (500 μM) could significantly up-regulate miR-25 expression in H9c2 embryonic rat ventricular myocytes [40]. Furthermore, the overexpression of miR-25 markedly reduced the oxidative stress-induced apoptosis in H9c2 cells by down-regulating mitochondrial calcium uniporter (MCU) which has been shown to control the Ca^2+^ flux through the inner mitochondrial membrane [40] (Figure 2).

#### Intimal hyperplasia

Thrombospondin-1 is known as a key factor in vascular smooth muscle cell (VSMC) migration after vascular injury. It has been reported that the expression of miR-25-5p beyond other miRNAs was significantly decreased in human VSMCs in response to a 6-hour thrombospondin-1 treatment [41] (Table 1). This study may suggest that miR-25-5p and other altered microRNAs might contribute to the development of atherosclerosis and intimal hyperplasia.

#### Intracranial aneurysm

Interestingly, a clinical cohort study enrolling 40 patients with intracranial aneurysm (IA), 20 healthy volunteers and an independent validation cohort including 93 IA patients reported that increased plasma miR-25 level might be a potential biomarker for intracranial aneurysm [42] (Table 1).

#### Pulmonary arterial hypertension

Pulmonary arterial hypertension (PAH) is an obstructive, arterial vasculopathy characterized by excessive pulmonary artery smooth muscle cell (PASMC) proliferation and migration [43]. It leads to vascular stiffening, vasoconstriction, mitochondrial and metabolic dysfunction and finally right ventricular failure [43]. A study proved that overexpression of miR-25 in human PASMC samples resulted in decreased expressions of mitochondrial calcium uniporter (MCU) and cAMP response element binding protein (CREB1). These gene expression alterations resulted in mitochondrial and metabolic dysfunction and a cancer-like phenotype with apoptosis resistance [43] (Table 1) (Figure 2). The downregulation of MCU by miR-25 has also been found in cardiomyocytes by Pan et al. [40] (Table 1) (Figure 2).

### Diabetes mellitus

Diabetes mellitus is a heterogeneous chronic metabolic disorder characterized by hyperglycemia as a common feature resulting from impaired insulin secretion, insulin resistance, or both [44]. Type-2 diabetes mellitus (T2DM) accounts for more than 85% of all diabetes cases, and its incidence is continuously rising worldwide [44]. It has been reported that high-fat diet and streptozotocin-induced T2DM lead to increased pancreatic miR-25 expression and decreased mRNA expression of its direct target insulin stabilizing polypyrimidine tract binding protein 1 (PTBP1) which resulted in decreased insulin translation and secretion [45] (Table 1) (Figure 2). Moreover, a clinical study enrolling two T1DM cohorts (n=275 for European T1DM children and 129 for Danish T1DM children) and one control group (n=151) reported that serum miR-25 level was significantly increased in T1DM children and was inversely correlated with residual beta cell function [46] (Table 1).

### Chronic kidney disease

Chronic kidney disease is often associated with tubulointerstitial fibrosis leading to progressive functional deterioration [20]. It has been demonstrated that all members of the miR-106b-25 cluster including miR-106b, miR-93 and miR-25 were significantly down-regulated after 48 h TGF-β1 treatment (5 ng/ml) in human kidney proximal tubular epithelial cell line (HK-2) [20] (Table 1). Bioinformatics analysis identified the TGF-β type-II receptor as a potential target of the miR-106b-25 cluster. Indeed, miR-106b transfection resulted in decreased expression of TGF-β type II receptor in HK-2 cells [20] (Table 1). Hence, the repression of miR-25 and miR-106b may be a key factor in the development of TGF-β1-mediated fibrosis both in the kidney and the heart [32] (Table 1).

Diabetes mellitus is one of the leading causes of end-stage renal disease [47]. NADPH oxidase-derived superoxide seems to play a key role in hyperglycemia-induced oxidative stress in diabetic nephropathy [48]. MiR-25 expression was significantly reduced and NOX4 mRNA and protein levels were increased in the kidney of streptozotocin-induced diabetic rats and high glucose-treated mesangial cells [45] (Table 1). Furthermore, mesangial cells transfected with antagomiR-25 showed significantly increased NOX4 mRNA and protein levels [48] (Table 1) (Figure 2). These results are in line with our findings in the hearts of hypercholesterolemic rats with increased cardiac tissue oxidative stress and diastolic dysfunction in the presence of decreased miR-25 expression [39] (Table 1) (Figure 2). Therefore, the miR-25-NOX4-oxidative stress axis seems to play a common role in kidney and heart disease.

IgA nephropathy is the most common primary glomerulonephritis leading to end-stage renal disease [49]. A clinical cohort study recruiting 3 control and 9 IgA nephropathy patients showed that miR-25 was significantly increased in the urinary sediment derived mainly from urinary erythrocytes of IgA nephropathy patients [49].

### Non-cancerous nervous system diseases

#### Cerebral ischemia/reperfusion injury

Cerebral ischemia is a condition when blood and oxygen supply to the brain tissues is insufficient [50, 51]. Rapid reperfusion is still the gold standard therapy for cerebral ischemia and other types of ischemic organ damages including e.g., ischemic stroke, myocardial infarction and organ transplantations [50]. However, reperfusion causes further tissue injuries due to increased oxidative/nitrative stress leading to mitochondrial dysfunction, lipid peroxidation, protein oxidation, DNA damage and finally cell death [38]. MiR-25 was repressed in a model of 48-h oxygen-glucose deprivation and 48-h reoxygenation in human neuroblastoma SH-SY5Y and IMR-32 cells [50] (Table 2). Furthermore, overexpression of miR-25 protected cells against apoptosis induced by oxygen-glucose deprivation/reoxygenation possibly through the Fas/FasL pathway [50] (Table 2) (Figure 2). This protective effect of miR-25 against apoptosis in human neuroblastoma SH-SY5Y and IMR-32 cells are in concordance with the findings of Pan et al. reported in H9c2 embryonic rat ventricular myocytes [40, 50] (Table 1-2) (Figure 2). Interestingly, 10-Hz repetitive transcranial magnetic stimulation was reported to improve proliferation of adult neural stem cells 7 days after focal cerebral ischemia in the subventricular zone in rats [52] (Table 2) Moreover, the beneficial effect of repetitive transcranial magnetic stimulation was associated with the overexpression of miR-25 and repression of its direct target p57 (CDKN1C). It is a member of the cyclin-dependent kinase (CDK) inhibitors blocking the cell cycle in G1/S phase [49] (Table 2) (Figure 2). Interestingly, a study related to CNS development in zebrafish embryos has also shown that scratch2 transcription factor could block the cell cycle re-entry by maintaining high levels of CDKN1C (p57) via the repression of miR-25 in postmitotic primary neurons [53] (Table 2) (Figure 2).

**Table 2:** The role of miR-25 in non-cancerous nervous system diseases

	Disease	Species and tissue or cell type	Alteration of miR-25 expression	Method for miR-25 detection	Target gene	Biological function	Method for target validation	Sample size in clinical studies	Ref.
1	Cerebral I/R	SH-SY5Y and IMR-32 cells (48 h OGD/ 48 h R)	up-regulation	qRT-PCR	Fas	apoptosis	**DLRA**, miR-25 TF, WB	N/A	[50]
2	Cerebral I/R	brain of male SD rats (7 days)	up-regulation	qRT-PCR	p57	cell cycle arrest	antagomiR-25, WB	N/A	[52]
3	CNS development	postmitotic primary neurons of zebrafish embryos	*down-regulation*	qRT-PCR	p57	cell cycle arrest	**DLRA**	N/A	[53]
4	SCA3/MJD	SCA/MJD model cells, 293T and SH-SY5Y cells	up-regulation	qRT-PCR	ATXN3	ataxia	**DLRA**	N/A	[51]
5	SCA3/MJD	human BS (Chinese)	*down-regulation*	qRT-PCR	N/A	N/A	N/A	35 SCA/MJD vs. 25 CONT	[54]
6	MS	human BP, T-cells (Italian)	*down-regulation* *(miR-106b-25 cluster)*	Agilent human miRNA microarray V2.0, qRT-PCR	N/A	N/A	N/A	12 MS vs 14 CONT	[56]
7	Schizophrenia	mouse hippocampus (8-10, 16-20 wk) human brain tissues	*down-regulation*	Agilent mouse miRNA microarray, qRT-PCR	SERCA2	calcium handling	miR-25 viral infection	17 schizophrenia vs. 22 CONT	[57]
8	Asthma bronchiale	human tracheal SMCs	*down-regulation*	miRNA array	KLF4	inflammation	WB, anti-miR-25 TF	N/A	[60]
9	CF	human BS (Australian)	up-regulation	Qiagen miRNA PCR array for serum and plasma	N/A	N/A	N/A	52 CF with liver disease50 CF without liver disease	[153]
10	Liver regeneration	male SD rats (6, 12, 24, 36 h)	up-regulation	Agilent rat miRNA array, V16, 8×15k	RB1, KAT2B	cell cycle arrest	**LRA**, IH, WB	N/A	[149]

Abbreviations: BP: blood plasma, BS: blood serum, CF: cystic fibrosis, CNS: central nervous system, CONT: control, DLRA: dual luciferase reporter assay, h: hour, IF: immunofluorescence, IH: immunohistochemistry, I/R: ischemia/reperfusion, LRA: luciferase reporter assay, KO: knock out, MS: multiple sclerosis, OE: overexpression, OGD: oxygen glucose deprivation, R: reoxygenation, SCA/MJD: Spinocerebellar ataxia type 3/Machado-Joseph Disease, SD: Sprague Dawley, SMCs: smooth muscle cells, TF: transfection, TG: transgene, WB: Western blot, wk: week. The abbreviations in bold are considered as gold standard methods for miRNA target validation.

#### Spinocerebellar ataxia type 3

Spinocerebellar ataxia type 3/Machado Joseph disease (SCA3/MJD) is the most common type of inherited spinocerebellar ataxia forms [48], and its symptoms include cerebellar ataxia, spasticity, parkinsonism, dystonia, eye movement disorders, sensory loss, muscle weakness, fasciculation, etc. [54]. SCA/MJD is also known as a polyglutamine (polyQ) disease caused by glutamine-encoding CAG nucleotide expansions within endogenous human genes resulting in an abnormal polyQ tract in the polyQ-expanded mutant ataxin-3 protein [48]. This abnormal ataxin-3 protein aggregates in the nucleus and adjacent areas of the affected neurons exacerbating cell death [51]. Overexpression of miR-25 was found to suppress apoptosis in SCA/MJD model cells possibly by the posttranscriptional reduction of polyQ-expanded ataxin-3 protein levels [51] (Table 2) (Figure 2). Indeed, the same research group reported in a clinical cohort study that serum level of miR-25 was significantly lower in SCA3/MJD patients (n=35) as compared to healthy controls (n=25) [51] (Table 2). Moreover, serum miR-25 level was significantly decreased in SCA3/MJD patients with a course of disease more than 6 years as compared to those patients with shorter disease course [51].

#### Multiple sclerosis

Multiple sclerosis (MS) is an inflammatory and subsequently degenerative disease of the central nervous system (CNS). It is defined by focal demyelinated lesions in the white matter of the brain and spinal cord [55]. A clinical study enrolling 12 MS relapsing–remitting patients in stable condition and 14 healthy controls revealed that members of the miR-106b-25 cluster were down-regulated in CD4^+^CD25^high^CD^127dim/−T^ regulatory cells of MS patients [56] (Table 2).

#### Schizophrenia

Schizophrenia is a chronic severe neuropsychiatric disorder with strong genomic and environmental risk factors. The expression of miR-25, a transcriptional regulator of SERCA2 was downregulated in a mouse model of schizophrenia [57] (Table 2) (Figure 2). The 22q11 deletion syndrome (22q11DS) is one of the strongest known genetic risks for schizophrenia [54]. A mouse model of 22q11DS had an age-dependent increase in hippocampal long-term potentiation (LTP), a form of synaptic plasticity needed in learning and memory [57, 58]. In this mouse model of schizophrenia, the expression of SERCA2 is increased resulting in elevated loading of the endoplasmic reticulum with Ca^2+^ and enhanced neurotransmitter release as well as increased LTP [57, 58]. A study has found that haploinsufficiency of DGCR8; a miRNA biogenesis gene in the 22q11DS disease-critical region led to synaptic SERCA2 overexpression and increased LTP [57]. Moreover, SERCA2 was elevated in human brain samples with schizophrenia [57] (Table 2) (Figure 2).

#### Neurotoxicity by dioxins

Acetylcholinesterase (AChE) plays a central role in cholinergic neurotransmission in central and peripheral nervous systems by hydrolysation of the neurotransmitter, acetylcholine [56]. Dioxins were shown to decrease AChE expression directly in neuroblastoma cells and immune cells by transcriptional regulation via aryl hydrocarbon receptor and by post-translational regulation via microRNAs including miR-25 [59]. Proposed mechanisms of dioxin toxicity are reviewed in detail by Xie *et al.* [59].

### Asthma bronchiale

A study using human tracheal smooth muscle cells revealed that miR-25 was significantly down-regulated after exposing cells to pro-inflammatory cytokines including IL-1β, TNF-α, and IFN-γ [60]. In this study, the repression of miR-25 resulted in the overexpression of its direct target Krüppel-like factor 4 (KLF4). KLF4 is known as an inhibitor of smooth muscle-specific gene expression and mediator of inflammation [60] (Table 2) (Figure 2).

### Cancerous diseases

#### Cancerous nervous system diseases

#### Glial tumors

**Figure 3:**
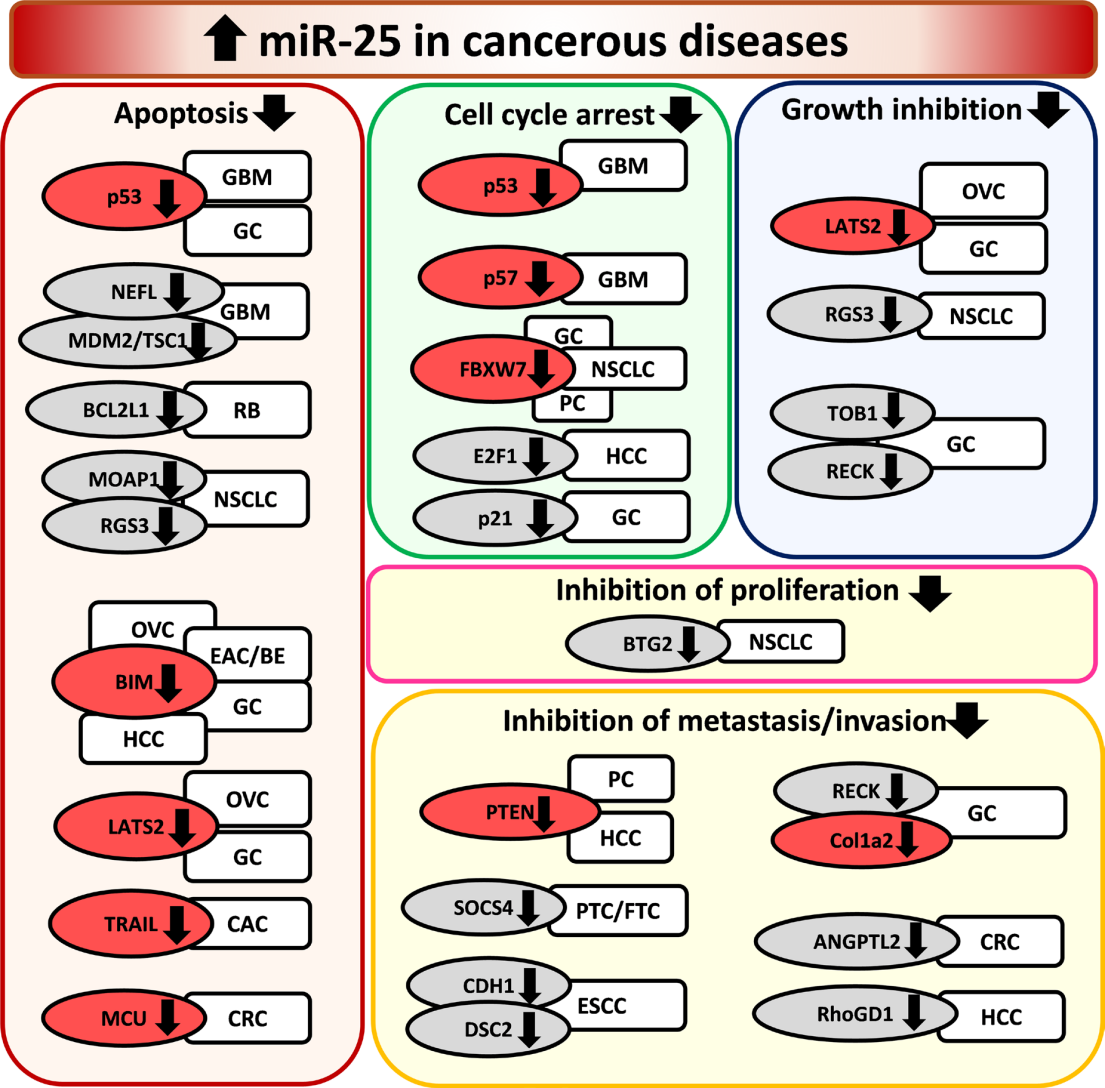
Overexpression of miR-25 in cancerous diseases EAC/BE: esophageal adenocarcinoma/Barrett esophagus, ESCC: esophageal squamous cell carcinoma, CAC: cholangiocarcinoma, CRC: colorectal cancer, GC: gastric cancer, GBM: glioblastoma multiforme, HCC: hepatocellular carcinoma, NSCLC: non-small cell lung carcinoma, OVC: ovarian cancer, PC: prostate cancer, PTC/FTC: papillary thyroid carcinoma/follicular thyroid carcinoma, RB: retinoblastoma. Gene symbols in bubbles are targets of miR-25 in multiple organs/diseases. Genes in red bubbles are targets of miR-25 in multiple diseases.

Astrocytoma originating from astrocytic glial cells is the most common type of primary tumor type in the central nervous system [61]. Anaplastic astrocytoma (AA) is a high-grade malignant glioma (grade III according to the WHO classification) developing from low-grade diffuse astrocytoma (DA; grade II) and progressing into glioblastoma of grade IV [61]. Glioblastoma multiforme is the most malignant and aggressive form of gliomas showing a median survival time of 15 months after standard therapy [62]. Few studies have investigated the role of miR-25 in the development of gliomas [62–66] (Table 3). Most of them indicate that miR-25 is overexpressed, and behave as an oncomiR during the development of gliomas [62, 63, 65, 66] (Table 3). These results suggest that miR-25 has mRNA targets directly or indirectly regulating pathways related to cell cycle or cell death. Zhang *et al*. reported that miR-25 was overexpressed in more than 90% of human glioma tissues (grade II-IV) and 60% of human glioma cell lines [62]. Furthermore, miR-25 has been shown to increase glioma cell proliferation by directly targeting the CDK inhibitor type 1C (CDKN1C or p57) resulting in an increase of S/M phase cells and a decrease of G0/G1 phase cells [62] (Table 3) (Figure 3). The miR-25 and CDKN1C (p57) axis seem to play a central role in the regulation of cell cycle re-entry not only in glioblastoma and cancerous cell proliferation but also in healthy conditions and after ischemia/reperfusion injury as mentioned in the previous section [52, 53, 62]. In accordance with the findings of Zhang *et al.*, another study has found that miR-25 was overexpressed in human astrocytoma samples and glioblastoma cell lines leading to tumor growth and invasion by directly targeting neurofilament light polypeptide (NEFL) which was an inhibitor of the mammalian target of rapamycin (mTOR) cell proliferation pathway [65] (Table 3) (Figure 3). Interestingly, a network analysis study investigating transcription factors, miRNAs and their target genes in human anaplastic astrocytoma reported that miR-25 might target tumor suppressor p53 and it could be regulated by another tumor suppressor, the phosphatase and tensin homolog (PTEN) [61] (Table 3) (Figure 3). In contrast, only one study has reported that overexpression of miR-25 could suppress glioblastoma growth *in vivo* and *in vitro* by a p53 tumor suppressor-dependent mechanism [64] (Table 3) (Figure 3). In this study, miR-25 was identified as a miRNA repressed indirectly by p53 through the transcriptional regulators of the *P53* gene, E2F1 (also called retinoblastoma binding protein-3) and MYC [64] (Table 3) (Figure 3). In addition, overexpression of miR-25 resulted in the downregulation of its direct targets MDM2 and TSC1. Both targets are negative regulators of the p53 tumor suppressor and the mTOR cell proliferation pathway, respectively [64]. In that article, the authors speculated on that overexpression of miR-25 could stabilize p53 tumor suppressor expression through activation of mTOR pathway by targeting TSC1. However, this finding is controversial to the results of Peng et al. [64, 65].

**Table 3:** The role of miR-25 in cancerous nervous system diseases

	Disease	Species and tissue or cell type	Alteration of miR-25 expression	Method for miR-25 detection	Target gene	Biological function	Method for target validation	Sample size in clinical studies	Ref.
1	GBM	human brain tumor tissue (American)	up-regulation	Agilent human miRNA microarray version 1, qRT-PCR	N/A	N/A	N/A	24 CNS tumors vs. 8 CONT	[66]
2	GBM	human GBM sample (Chinese), GMB cell lines	up-regulation	qRT-PCR	NEFL	apoptosis	**LRA**, WB	44 GBM vs. 20 CONT	[65]
3	GBM	human GBM sample (Chinese), human GBM cell lines	up-regulation	qRT-PCR	p57	cell cycle arrest	**LRA**, miR-25 TF	35 GBM vs. 5 CONT	[62]
4	GBM	human GBM sample (dataset)	up-regulation	dataset analysis	p53	apoptosis, cell cycle arrest	network analysis	N/A	[61]
5	GBM	human GBM cell lines, mice (35 days)	up-regulation	Nanostring assay, qRT-PCR	MDM2, TSC1	apoptsosis	**LRA**, miR-25 TF, WB	N/A	[64]
6	RB	human RB sample (Chinese)	up-regulation	Agilent human miRNA microarray 2k	Bcl2L1	apoptosis	exp. validated databases	3 RB vs. 3 CONT	[67]

Abbreviations: CONT: control, DLRA: dual luciferase reporter assay, GBM: glioblastoma multiforme, IF: immunofluorescence, IH: immunohistochemistry, LRA: luciferase reporter assay, KO: knock out, OE: overexpression, RB: retinoblastoma, TF: transfection, TG: transgene, WB: Western blot. The abbreviations in bold are considered as gold standard methods for miRNA target validation.

#### Retinoblastoma

Retinoblastoma (RB) is a typical malignant tumor appearing in children [67]. A clinical pilot study with a very limited sample size enrolling 3 healthy and 3 RB retina samples has reported that miR-25 was significantly overexpressed in RB [67] (Table 3) (Figure 3). After miRNA-target analysis using experimentally validated databases and pathway enrichment analysis, the apoptosis regulator BCL2L1 seemed to be a potentially important target of miR-25 in RB [67] (Table 3) (Figure 3).

### Lung cancer

#### Non-small cell lung cancer

**Figure 4:**
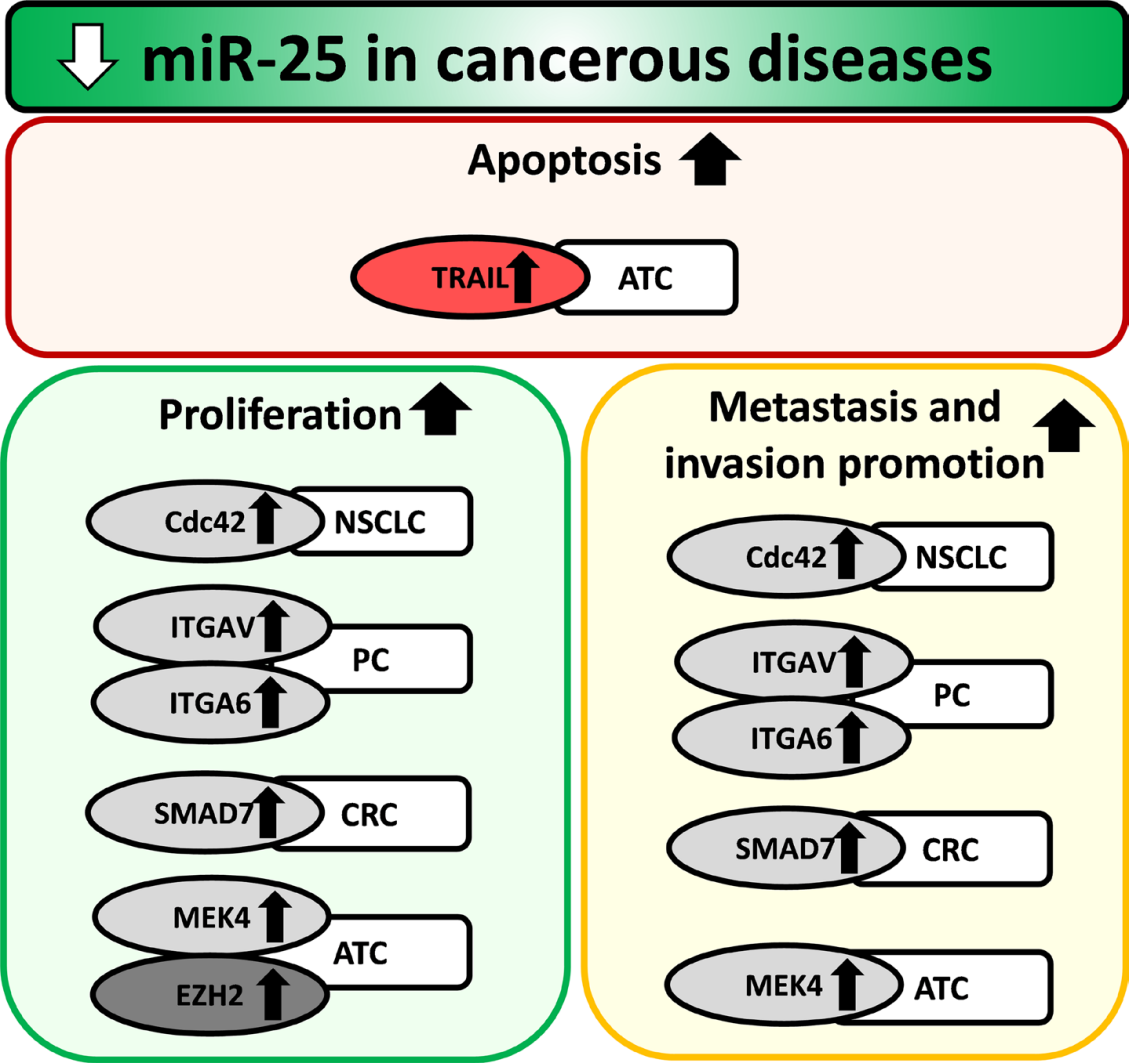
Repression of miR-25 in cancerous diseases ATC: anaplastic thyroid cancer, CRC: colorectal cancer, NSCLC: non-small cell lung carcinoma, PC: prostate cancer. Gene symbols in bubbles are targets of miR-25 in multiple organs/diseases. Gene in red bubble is a target of miR-25 in multiple diseases.

Lung cancer is the leading cause of tumor-related mortality worldwide [68]. Non-small cell lung cancer (NSCLC) accounts for 80-85% of all lung cancer cases, and its 5-year survival rate is about 15% [68]. Savita et al. reported that the overexpression of the miR-106b-25 cluster could directly suppress the ubiquitin ligase β-TRCP2 gene expression leading to decreased Snail degradation in H1299 non-small cell lung cancer cells [69] (Table 4) (Figure 3). Snail has been reported to positively regulate cell adhesion and migration as well as invasion [70, 71]. Xiang et al. demonstrated that the oncogenic effect of miR-25 is partially due to direct targeting and repressing F-box and WD repeat domain-containing 7 (FBXW7 also known as FBW7) in human NSCLC tissue samples and cell lines [68] (Table 4) (Figure 3). FBXW7 is a putative tumor suppressor in human tumorigenesis due to its ability to recognize and bind target proteins for ubiquitination and degradation [68]. Other studies suggested that the overexpression of miR-25 could reduce apoptosis by different mechanisms in human NSCLC cells and cell lines [72, 73] (Table 4). Wu et al. reported that miR-25 directly targeted the modulator of the apoptosis 1 (MOAP1) gene which was a Bax-associated protein containing BH3-like motif and mediating caspase-dependent apoptosis [72] (Table 4) (Figure 3). Chen et al. found that overexpression of miR-25 directly repressed the regulator of G protein signaling 3 (RGS3)gene which potentially could play a role in apoptosis as a cancer suppressor [73] (Table 4) (Figure 3). He et al. reported that radiotherapy-resistant NSCLC human tissues overexpressed miR-25 as compared to radiosensitive or non-cancerous tissues [74] (Table 4). In addition, miR-25 overexpression correlated negatively with its direct BTG anti-proliferation factor 2 (BTG2) expression (Table 4) (Figure 3). BTG2 has been shown to inhibit cell proliferation and invasion by repressing cyclin D1, matrix metalloproteinase-1 and metalloproteinase-2 in human lung cancer cells [74]. Interestingly, down-regulation of miR-25 by antagomiR-25 treatment was shown to inhibit NSCLC cell proliferation and induce G1 cell cycle arrest possibly through indirect down-regulation of the cell division cycle 42 (CDC42) gene [75] (Table 4) (Figure 4).

**Table 4:** The role of miR-25 in lung cancer

	Disease	Species and tissue or cell type	Alteration of miR-25 expression	Method for miR-25 detection	Target gene	Biological function	Method for target validation	Sample size in clinical studies	Ref.
1	NSCLC	H1299 cells	up-regulation of miR-106b and miR-93	qRT-PCR	beta-TRCP2	ubiquitination	**LRA**	N/A	[69]
2	NSCLC	human NSCLC tissue (Chinese), human NSCLC cell lines	up-regulation	qRT-PCR	FBXW7	cell cycle arrest	**LRA**, miR-25 TF, WB	16 NSCLC vs. 16 CONT	[68]
3	NSCLC	human BP (Chinese), human NSCLC cell lines	up-regulation	qRT-PCR	MOAP1	apoptosis	**LRA**, miR-25 TF, MOAP1 OE or KO, anatgomiR-25 *in vivo*	81 NSCLC vs. 41 CONT	[72]
4	NSCLC	human NSCLC tissue (Chinese), human NSCLC cell line	up-regulation	qRT-PCR	RGS3	apoptosis	**DLRA**, WB	35 NSCLC vs. ADJNTT	[73]
5	NSCLC	human NSCLC tissue (Chinese), human NSCLC cell line	up-regulation	qRT-PCR	BTG2	proliferation inhibitor	**LRA**, antagomiR-25 and miR-25 TF *in vitro*, WB	60 NSCLC vs. 32 CONT	[74]
6	NSCLC	human NSCLC tissue (Chinese), human NSCLC cell line, female nude mice (35 days)	*down-regulation*	qRT-PCR	CDC42	proliferation	**LRA**, antagomiR-25 TF *in vivo*, CDC42 OE, WB	11 NSCLC vs. ADJNTT	[75]
7	NSCLC	human BS (American, Chinese)	up-regulation	Taqman low density array, qRT-PCR	N/A	N/A	N/A	221 NSCLC vs. 161 CONT (56 benign nodules)	[77]
8	NSCLC	human NSCLC tissue, human BP (Chinese)	up-regulation	qRT-PCR	N/A	N/A	N/A	100 female NSCLC (non-smoking)	[63]
9	SCLC	human SCLS tissue (Chinese), human SCLC cell lines	up-regulation	qRT-PCR	CDK2	proliferation	**LRA**, anatgomiR-25 *in vitro*, WB	9 SCLC vs. ADJNTT	[80]

Abbreviations: ADJNTT: adjacent non-tumorous tissue, BP: blood plasma, CONT: control, DLRA: dual luciferase reporter assay, IF: immunofluorescence, IH: immunohistochemistry, LRA: luciferase reporter assay, KO: knock out, NSCLC: non-small cell lung carcinoma, OE: overexpression, SCLC: small cell lung carcinoma, TF: transfection, TG: transgene, WB: Western blot. The abbreviations in bold are considered as gold standard methods for miRNA target validation.

REV3Lp is the catalytic subunit of DNA polymerase zeta playing a crucial role in genome stability in mammalian cells. A study investigating the polymorphism of REV3Lp in lung cancer susceptibility in Chinese Han population (n=500 lung cancer patients and 517 cancer-free control) revealed that (3’UTR) 460 T>C single nucleotide polymorphism (rs465646) showed a strong association with lung cancer development [76]. The T allele demonstrated a stronger binding affinity for miR-25 and miR-32 which could down-regulate the endogenous tumor suppressor REV3L [76]. Furthermore, an ethnically diverse multicenter case-control study recruiting 221 NSCLC patients, 161 controls and 56 patients with benign nodules from China and America reported that serum levels of miR-25 and other four miRNAs (miR-483-5p, miR-193a-3p, miR-214 and miR-7) were significantly elevated irrespective of ethnicity groups [77] (Table 4). A clinical study enrolling 100 Chinese female non-smoking lung adenocarcinoma patients found that increased plasma miR-25 levels positively correlated with the mortality rate, advanced disease stage, regional and distant metastasis at diagnosis as well as epithelial growth factor receptor (EGFR) mutation status [63] (Table 4). Another clinical study investigating circulating miRNA levels in response to chemotherapy or operation in Russian lung cancer patients (n=23, T_1-3_N_0-3_M_0_) showed that the plasma concentration of miR-25 did not change 15 days after surgery or within 30 days after completing two courses of paclitaxel-carboplatin chemotherapy as compared to that before the respective intervention [78]. In another clinical study enrolling 148 Chinese patients with histologically proven advanced or metastatic lung adenocarcinoma (stage IIIB or IV) and showing a complete or partial response to first-line therapy with the antifolate agent pemetrexed plus platinum of 4-6 cycles, were divided into an observation *versus* pemetrex maintenance group [79]. In the pemetrex maintenance group, significantly elevated serum miR-25 levels were negatively correlated with progression-free survival time [79]. In contrast, serum miR-25 levels failed to correlate with progression-free survival time in the observation group [79].

#### Small cell lung cancer

Small cell lung cancer (SCLC) accounts for approximately 15% of all new lung cancer cases and has unique clinical and histological characteristics [80]. Zhao et al., similarly to that in NSCLC samples, reported the overexpression of miR-25 in human SCLC tissue samples and cell lines [80]. Interestingly, down-regulation of miR-25 by antagomiR-25 treatment was shown to inhibit SCLC cell proliferation and induced the cell cycle arrest in the G1 phase possibly through the down-regulation of the cell cycle-related proteins cyclin E2 and CDK2. Cyclin E2 was shown to be a direct target of miR-25 in this study [80] (Table 4) (Figure 3).

### Breast cancer

Breast cancer ranks the first among female cancers and is the second leading cause of death in women worldwide [81, 82]. Breast cancer is a heterogeneous disease entity, and numerous classification systems have been developed on the basis of histopathological and molecular genetic features or the appearance in imaging studies such as mammography or MRI [83]. From the point of view of therapy, both the stage/extent of the tumor and mentioned special features contribute to therapeutic decision. Breast cancer has always been managed more or less on an individual basis; the recent advances in precision medicine made a great impact on the management of breast cancer. Plenty of studies have examined the correlation between miRNA and mRNA expression in breast cancer and the role of miRNA expression profile in prognosis, but no clear conclusion has yet been reached [84]. The vast majority of studies investigating the role of miR-25 in the development of breast cancer have found that miR-25 was overexpressed in human breast cancer tissues and was elevated in the serum of patients [82, 84–90] (Table 5) (Figure 3). Hu et al. demonstrated in a two-stage case-control analysis that serum levels of miR-25, miR-16, miR-222 and miR-324-3p were significantly upregulated as compared to those other miRNAs (miR-191 and miR-484) used as endogenous control in Chinese breast cancer patients [85] (Table 5). Wu reported that miR-25 expression was significantly higher in breast cancer patients based on data of 683 breast cancer tissues when compared to that in 87 normal breast tissues according to The Cancer Genome Atlas [83]. Interestingly, another clinical study enrolling 240 Norwegian early breast cancer patients showed strong and significant associations between the overexpression of miR-25, miR-18a/b, miR-106b, and miR-505 with high proliferation, estrogen receptor negativity and cytokeratin 5/6 positivity of cancer [86] (Table 5). Moreover, using dataset analysis Farazzi et al. reported that miRNA families might control subtype-specific pathways in breast cancer and miR-25 showed high miRNA regulatory activity in triple-negative and basal-like subtypes of breast cancer [84]. Another study using 21 surgical breast cancer specimens revealed that increased expression of miR-25 was associated with high Ki-67 (a marker of cell proliferation) expression and HER2, ER and PR positivity [88] (Table 5). Interestingly, a study enrolling 76 breast cancer patients reported that overexpression of miR-25^*^, miR-142-3p, miR-505^*^, miR-1248, miR-181a-2^*^, and miR-340^*^ could discriminate between tumor samples from BRCA1/2 mutation carriers and non-carriers [90]. A BRCA1/2A preclinical mechanistic study reported that down-regulation of miR-25 by isoliquiritigenin resulted in increased autophagic cell death by overexpression of ULK1 and induced chemosensitization in MCF7/ADR breast cancer cells [91] (Table 5) (Figure 3). Another study comprehensively analyzed tumor tissue miRNA expression and patient survival collecting data from the Cancer Genome Atlas (TCGA) which contains miRNA sequencing and overall survival datasets of 759 breast cancer patients [83, 89]. Surprisingly, this study found that increased expression of miR-25 predicted improved breast cancer survival [89].

**Table 5:** The role of miR-25 in breast, ovarian and prostate cancer

	Disease	Species and tissue or cell type	Alteration of miR-25 expression	Method for miR-25 detection	Target gene	Biological function	Method for target validation	Sample size in clinical studies	Ref.
1	BC	human BS (Chinese)	up-regulation	Solexa sequencing, Taqman low density array, qRT-PCR	N/A	N/A	N/A	24 BC vs. 48 CONT	[85]
2	BC	human BC tissue, human BS (Chinese)	up-regulation	Solid sequencing, qRT-PCR	N/A	N/A	N/A	88 BC vs. 60 CONT	[82]
3	BC	human BC tissue (dataset)	up-regulation	qRT-PCR and dataset analysis	N/A	N/A	N/A	683 BC vs. 87 CONT	[91]
4	BC	human BC tissue (Norwegian)	up-regulation	qRT-PCR	N/A	N/A	N/A	240 BC (T1,2N0M0)	[86]
5	BC	human BC tissue (Japanese)	up-regulation	Qiagen Human Cancer microRNA PCR Array system	N/A	N/A	N/A	21 BC	[88]
6	BC	human BC cell line, NOD/SCID mice	up-regulation	Affymetrix miRNA 3.0 array, qRT-PCR	ULK1	autophagy	**LRA**, WB	N/A	[81]
7	OVC	human OVC cell lines, ovarian surface epithelial cells	up-regulation	qRT-PCR	BIM	apoptosis	**LRA**, TF of miR mimics and antagomiR-25 *in vitro*, WB	N/A	[95]
8	OVC	human OVC cells, normal ovarian epithelial cell line	up-regulation	qRT-PCR	LATS2	apoptosis, growth inhibition	**LRA**, miR-25 mimics and antagomiR-25 TF, WB	N/A	[93]
9	OVC	human OVC tissue (Chinese)	up-regulation	qRT-PCR	N/A	N/A	N/A	86 OVC vs. ADJNTT	[96]
10	OVC	human BS (German, 21 months) human OVC cell lines	*down-regulation*	qRT-PCR	N/A	N/A	N/A	180 OVC vs. 66 CONT	[97]
11	OVC	human BS (Irish)	*down-regulation*	qRT-PCR	N/A	N/A	N/A	25 OVC vs. 25 CONT	[92]
12	OVC	human BP (American, 15 months)	*down-regulation*	qRT-PCR	N/A	N/A	N/A	14 OVC	[98]
13	PC	human PC tissue (American), MCM7 TG mice, nude mice (7 wk), human PAC cell lines	up-regulation of the miR-106b-25 cluster and MCM7	qRT-PCR	PTEN	metastasis and invasion inhibitor	**DLRA**, TF *in vitro*, WB	177 PC vs. ADJNTT	[8]
14	PC	human PC cell lines	up-regulation of the miR-106b-25 cluster and MCM7	qRT-PCR	PTEN	metastasis and invasion inhibitor	no	N/A	[102]
15	PC	human PC tissue, human PC cell lines, hypoxic neural crest cells	up-regulation of the 106b-25 cluster	qRT-PCR	PTEN	metastasis and invasion inhibitor	anatgomiR-25 or miR-25 TF	9 PC	[101]
16	PC	prostate neuroendocrine cells, p53 knock down PC cells	up-regulation	qRT-PCR	FBXW7	cell cycle arrest	RNA interference	N/A	[103]
17	PC	human PC tissue (Brazilian)human PC cell lines	up-regulation	qRT-PCR	N/A	N/A	N/A	63 PC	[104]
18	PC	human osteotropic PC cell lines, zebrafish embryos	*down-regulation*	qRT-PCR	ITGA6, ITGAV	metastasis and invasion	**LRA**, miR-25 TF	N/A	[100]
19	PC	human BP (Caucasian)	*down-regulation*	qRT-PCR	N/A	N/A	N/A	68 PC vs. 79 CONT	[106]

Abbreviations: ADJNTT: adjacent non-tumorous tissue, BP: blood plasma, BS: blood serum, BC: breast cancer, DLRA: dual luciferase reporter assay, IF: immunofluorescence, IH: immunohistochemistry, LRA: luciferase reporter assay, KO: knock out, OE: overexpression, OVC: ovarian carcinoma, PC: prostate cancer, TF: transfection, TG: transgene, WB: Western blot. The abbreviations in bold are considered as gold standard methods for miRNA target validation.

### Ovarian cancer

Ovarian cancer is the most common cause of gynecological malignancy-related mortality among women [92]. Epithelial ovarian cancer (EOC) accounts for approximately 90 % of ovarian tumors, including serous adenocarcinoma, endometrial adenocarcinoma and clear cell carcinoma [93]. Most cases are not diagnosed at an early stage [89]. Therefore, the 5-year survival rate is still poor despite advances in the diagnosis and chemotherapy [93, 94]. Studies investigating the expression of miR-25 in epithelial ovarian cancer cell lines, tumorous tissues and serum from patients provided controversial findings. A preclinical study demonstrated that miR-25 was overexpressed in human ovarian cancer cells and cell lines (SKOV3, OVCAR3, OVCAR5, and A2780) and ovarian surface epithelial cells (OSE) [92] (Table 5) (Figure 3). In this study, repression of miR-25 in ovarian cancer cells enhanced apoptosis by directly targeting Bim (also known as BCL2L11) [95]. Bim is also known as a direct activator of Bax and neutralizer of Bcl2-like molecules [95] (Table 5) (Figure 3). Another preclinical study showed that expression of miR-25 was increased in human ovarian cancer cells (OVCAR3, SKOV3, ES-2) [90] (Table 5) (Figure 3). In this study, inhibition of miR-25 could suppress proliferation, migration, and invasion of ovarian cancer cells by directly targeting large tumor suppressor 2 (LATS2) [93] (Table 5) (Figure 3). A clinical cohort study enrolling 86 ovarian cancer patients reported that the increased expression of miR-25 in EOC tissue was associated with advanced clinical stage, lymph node metastasis and shorter survival time indicating that miR-25 might be involved in carcinogenesis and metastasis of EOC [96] (Table 5). Moreover, another clinical cohort study recruiting 180 treated EOC patients and 66 healthy women from Germany showed that serum levels of miR-25 was down-regulated after a median follow-up time of 21 months [97] (Table 5). Interestingly, Langhe et al. demonstrated that four circulating microRNAs including miR-25-3p let-7i-5p, miR-122, and miR-152-5p were significantly down-regulated in serous ovarian carcinoma patients as compared to benign serous cystadenoma patients in a small sample size clinical study (n=25) [92] (Table 5). In other tissues, miR-25 is known to target WNT signaling, and AKT/mTOR pathways which have previously been found to play a role in ovarian carcinogenesis and chemoresistance as well [92] (Table 5). Moreover, Benson et al. reported that plasma miR-25 expression was significantly decreased in chemotherapy-resistant ovarian cancer patients, as compared to responders after 7 cycles of decitabine-carboplatin chemotherapy [98] (Table 5).

### Prostate cancer

Prostate cancer (PC) is the second most common cancer among men worldwide [99]. Both early detection and therapy have significantly improved recently, however, about 25% of patients develop metastases. Prostate carcinoma may develop to a clinically aggressive form, which is resistant to androgen deprivation therapy, develops metastases and expresses neuroendocrine markers [100, 101]. A study has proven that the concomitant overexpression of the miR-106b-25 cluster and its host gene minichromosome maintenance protein 7 (MCM7) cooperates in initiating prostate cancer by directly targeting the tumor suppressor phosphatase and tensin homologue (PTEN) gene both in prostate cancer cell lines and human prostate cancer specimens [8] (Table 5) (Figure 3). PTEN has been shown to suppress the PI3K/Akt pathway which activates a number of target proteins to promote nutrient uptake, protein synthesis, cell survival, cell proliferation, cell motility and angiogenesis [8]. Furthermore, a study by Liang et al. demonstrated that hypoxia-induced neuronal and neuroendocrine differentiation of neuronal crest cells and PC cells by inducing the miR-106b-25 cluster [101] (Table 5) (Figure 3). In this study, overexpression of the miR-106b-25 cluster resulted in the down-regulation of the tumor suppressor RE-1 silencing transcription factor (REST) [101] (Table 5) (Figure 3). Interestingly, REST has also been shown to be a negative regulator of PI3K/Akt pathway which is overactivated in PC [101]. Enterolactone produced by the metabolism of plant lignans by intestinal bacteria showed anti-proliferative effects in prostate cancer cell lines by repression of DNA licensing genes including MCM7 as well as the miR-106b-25 cluster, and overexpression of tumor suppressor gene PTEN [102] (Table 5). Interestingly, another mechanistic study by Li et al. showed that mutation of the p53 (TP53) tumor suppressor gene in prostate neuroendocrine cells could lead to overexpression of miR-25 similarly to the findings of Suh et al. in glioblastoma cells [64, 103] (Figure 3). This study by Li et al. demonstrated that the overexpression of miR-25 resulted in the repression of the E3 ubiquitin ligase FBXW7 leading to increased expression of its substrate Aurora kinase A (AURKA) which is a positive cell cycle regulator [103] (Table 5) (Figure 3). In contrast, Leite et al. found in advanced prostate cancer specimens (n=63) that the expression levels of several miRNAs including miR-25 significantly decreased during the progression of prostate cancer related to the transition from high grade prostate intraepithelial neoplasia to invasive adenocarcinoma, and the transition from localized to metastatic adenocarcinomas [104] (Table 5) (Figure 4). Moreover, Zoni et al. found that miR-25 was a negative regulator of the development of an invasive and metastatic phenotype in human prostate cancer cells by directly targeting the pro-invasive α_6_- and α_v_-integrins [100, 105] (Table 5). Indeed, a prospective clinical study enrolling 147 Caucasian age-matched patients with increased PSA levels and receiving needle biopsy to diagnose PC reported that decreased expression of miR-25-3p and other miRNAs correlated with increased malignancy [106] (Table 5). Interestingly, two clinical studies could not find any change in miR-25 expression in urine [107] or urine sediment samples [99] of prostate cancer patients.

### Thyroid cancer

Thyroid cancer represents the most common malignant tumor originating from endocrine organ including 1) well-differentiated papillary thyroid carcinoma (PTC, 80%), 2) follicular thyroid carcinoma (15%), 3) poorly differentiated thyroid carcinoma (<1%) and 4) anaplastic thyroid carcinoma (<2%) [108]. The role of miR-25 in the development of different histologic types of thyroid cancer seems to be controversial. Mei et al. published that a pro-inflammatory and carcinogenesis promoting cytokine, interleukin-23 (IL-23) could increase the expression of miR-25 in human thyroid cancer cell lines K1 (papillary type) and WRO (follicular type) promoting migration and invasion of thyroid cancer cells [109] (Table 6). This study also demonstrated that the overexpression of miR-25 directly reduced the expression of its direct target suppressor of cytokine signaling 4 (SOCS4) leading to increased IL-23 expression in human thyroid cancer cell lines and tissue samples [109] (Table 6) (Figure 3). A clinical cohort study enrolling 56 patients with primary PTC, 95 patients with benign thyroid nodules, and 10 age and gender-matched healthy controls from Northern China reported that plasma and tissue levels of miR-25-3p were significantly higher in PTC patients as compared to healthy controls or patients with benign thyroid nodules [108] (Table 6). In contrast, a research group found that miR-25 was repressed in anaplastic thyroid carcinoma using miRNA microarray [110] (Table 6) and qPCR for validation their results [111] (Table 6). They have also demonstrated that overexpression of miR-25 by transfection into ACT-1, 8505c and FRO human anaplastic thyroid cell lines could inhibit proliferation and colony formation by directly targeting the oncogene polycomb protein enhancer of zeste 2 (EZH2) [111] (Table 6) (Figure 4). Parallel with the aforementioned results, another research group has also found that the expression of miR-25 was decreased in anaplastic thyroid carcinoma [112] (Table 6) in human tissue samples and cell lines [113] (Table 6). Transfection of miR-25 into 8505c anaplastic thyroid cell line and Nthy-ori SV40-immortalised thyroid cell line could directly reduce the expression of survival-promoting mitogen-activated protein kinase 4 (MEK4) and tumor necrosis factor-related apoptosis inducing ligand (TRAIL) which could induce proliferation, migration, and invasion in tumor cells [113] (Table 6) (Figure 4).

**Table 6:** The role of miR-25 in thyroid cancer

	Disease	Species and tissue or cell type	Alteration of miR-25 expression	Method for miR-25 detection	Target gene	Biological function	Method for target validation	Sample size in clinical studies	Ref.
1	PTC/FTC	human PTC/FTC tissue (Chinese), human PTC/FTC cell lines	up-regulation	qRT-PCR	SOCS4	metastasis and invasion	**LRA**, RNA interference, WB	61 PTC/FTC vs. 44 CONT	[109]
2	PTC/FTC	human PTC/FTC tissue, human BP (Northern Chinese)	up-regulation	Agilent Human miRNA microarray kit 19.0	N/A	N/A	N/A	56 PTC vs. 10 CONT	[108]
3	ATC	human ATC tissue (Italian)	*down-regulation*	miRNA CHIP microarray, Northern blot	N/A	N/A	N/A	10 ATC vs. 10 CONT	[110]
4	ATC	human ATC tissue (French)human thyroid epithelial cell lines	*down-regulation*	qRT-PCR	EZH2	proliferation	**DLRA**, WB	N/A	[111]
5	ATC	human ATC tissue (Irish)	*down-regulation*	Multiplex stem-loop RT-PCR	N/A	N/A	N/A	1 CONT, 2 classic PTC, 1 insular PTC, 2 ATC, 1 lymphatic metastasis ATC, 1 vascular invasion ATC	[112]
6	ATC	human ATC cell line (Irish), human immortalized thyroid cell line	*down-regulation*	qRT-PCR	MEK4, TRAIL	proliferation, apoptosis	WB	N/A	[113]

Abbreviations: ATC: anaplastic thyroid cancer, BP: blood plasma, DLRA: dual luciferase reporter assay, IF: immunofluorescence, IH: immunohistochemistry, LRA: luciferase reporter assay, KO: knock out, OE: overexpression, PTC/FTC: papillary thyroid carcinoma/follicular thyroid carcinoma, TF: transfection, TG: transgene, WB: Western blot. The abbreviations in bold are considered as gold standard methods for miRNA target validation.

### Esophageal cancer

Esophageal cancer represents the eighth most common cancer and the sixth most common cause of tumorous death worldwide [114]. Esophageal cancer is developed from epithelial cells including two subtypes: 1) esophageal adenocarcinoma (EAC) and 2) esophageal squamous cell carcinoma (ESCC) [114]. Mir-25 was unequivocally reported to be increased in both subtypes of esophageal cancer tissues and serum/plasma samples of patients.

#### Esophageal adenocarcinoma

Esophageal adenocarcinoma (EAC) is an aggressive type of esophageal cancer with an overall 5-year survival rate of <20% [115]. Barrett's esophagus (BE) is a precursor abnormality of EAC [115]. In case of BE, the squamous epithelium of the esophagus is replaced by a metaplastic, columnar-lined epithelium originating from the gastroesophageal junction [115]. The progression of BE to adenocarcinoma follows a series of histologic evolvement with an increasing progression rate [115]. These histologic events include 1) non-dysplastic Barrett's metaplasia, 2) low-grade dysplasia (LGD), 3) high-grade dysplasia (HGD), and 4) adenocarcinoma [115]. Kan et al. demonstrated that increased level of miR-25 targeted and inhibited the translation of the pro-apoptotic Bim (also known as BCL2L11) in human EAC (OE-33) as well as metaplastic BE-derived cell lines (HEEpiC, QhTRT, ChTRT, GihTRT) and esophageal tissues (22 normal epithelia, 24 BE and 22 EAC) [116] (Table 7) (Figure 3). Zhang et al. had also reported increased miR-25 levels and decreased Bim expression in epithelial ovarian cancer cells similarly to the finding of the aforementioned study by Kan et al. in EAC [95, 116] (Figure 3). Moreover, the study by Kan et al. also reported that other elements of the miR-106b-25 cluster and its host gene, MCM7 were also overexpressed in EAC. These results are similar to the findings of Poliseno et al. in prostate cancer [8, 116] (Figure 3). Indeed, a clinical study investigating 35 normal epithelial, 34 BE, and 36 EAC tissues of American participants revealed that miR-25 and other miRNAs were overexpressed in BE and EAC samples with the most significant alteration in the BE metaplastic stage [115] (Table 7). Another clinical study using 119 tissue samples (24 normal/uninvolved mucosa samples from the distal esophagus of BE patients, 60 BE and 35 EAC) of Czech patients has also found that miR-25 and other miRNAs showed overexpression progressively in the sequence of normal mucosa, BE and EAC [117] (Table 7). Moreover, a clinical study enrolling Australian participants (19 healthy controls, 10 BE, and 18 locally advanced EAC patients) showed that a serum miRNA panel including miR-25 and other miRNAs (RNU6-1/miR-16-5p, miR-25-3p/miR-320a, let-7e-5p/miR-15b-5p, miR-30a-5p/miR-324-5p, miR-17-5p/miR-194-5p) had enhanced specificity and sensitivity over single miRNA ratios to distinguish EAC from controls and BE [118] (Table 7). In addition, a small sample size American study using 10 EAC (stage I-III) and 11 healthy control serum samples also verified that serum level of miR-25 was elevated in EAC [119] (Table 7).

**Table 7:** The role of miR-25 in gastrointestinal tumors

	Disease	Species and tissue or cell type	Alteration of miR-25 expression	Method for miR-25 detection	Target gene	Biological function	Method for target validation	Sample size in clinical studies	Ref.
1	EAC/BE	human EAC and BE cell lines, human EAC tissues (American)	up-regulation of the miR-106b-25 cluster and MCM7	Applied Biosystem Taqman MicroRNA assay (human), qRT-PCR	BIM	apoptosis	**LRA**, WB	22 CONT epithelia, 24 BE, 22 EAC	[116]
2	EAC/BE	human EAC and BE tissue (American)	up-regulation	Applied Biosystems’ real-time PCR-based TaqMan Human Micro-RNA Card Set v3.0 for 754 miRNAs	N/A	N/A	N/A	35 CONT epithelia, 34 BE, 36 EAC	[115]
3	EAC/BE	human EAC and BE tissue (Czech)	up-regulation	Affymetrix GeneChip miRNA 3.0 arrays	N/A	N/A	N/A	24 ADJNTT, 60 BE, 35 EAC	[117]
4	EAC/BE	human BS (Australian)	up-regulation	TaqMan OpenArray Human microRNA panel for 758 miRNAs	N/A	N/A	N/A	19 CONT, 10 BE, 18 EAC	[118]
5	EAC/BE	human BS (American)	up-regulation	Solexa deep sequencing for small RNAs, qRT-PCR	N/A	N/A	N/A	10 EAC vs. 11 CONT	[119]
6	ESCC	human ESCC cell lines, human ESCC tissue (Chinese)	up-regulation	qRT-PCR	CDH1	metastasis and invasion inhibitor	**LRA**, WB	N/A	[114]
7	ESCC	human ESCC cell lines, human ESCC tissue (Chinese)	up-regulation	qRT-PCR	DSC2	metastasis and invasion inhibitor	**LRA**, WB	124 ESCC	[121]
8	ESCC	human ESCC tissue (Northern Chinese)	up-regulation	qRT-PCR	N/A	N/A	N/A	5 ESCC	[122]
9	ESCC	human ESCC tissue, BS (Chinese)	up-regulation	qRT-PCR	N/A	N/A	N/A	20 ESCC vs. 20 CONT tissue, 194 ESCC vs. 94 CONT serum	[72]
10	ESCC	human BS (Chinese)	up-regulation	Taqman low density array, qRT-PCR	N/A	N/A	N/A	111 ESCC	[123]
11	ESCC	human BP (Japanese)	up-regulation	3D-Gene miRNA microarray, qRT-PCR	N/A	N/A	N/A	105 ESCC vs. 50 CONT	[120]
12	GC	human GC cell lines	up-regulation of the miR-106b-25 cluster and MCM7	miRNA microarray chips (V2) for 250 human miRNAs, qRT-PCR, Northern blot	BIM, p21	apoptosis cell cycle arrest	**LRA**, WB	N/A	[125]
13	GC	human GC cell lines	up-regulation of the 106b-25 cluster and MCM7	qRT-PCR	Col1a2, p53	metastasis and invasion inhibitor, apoptosis, cell cycle arrest	antagomiR-25 TF, IF	N/A	[127]
14	GC	human GC tissue and human BP (Chinese), human GC cell lines, nude mice	up-regulation	qRT-PCR	TOB1	growth inhibitor	**LRA**, antagomiR-25 TF, WB	103 GC vs. 80 CONT	[126]
15	GC	human GC tissue (Chinese, 80 months), human GC cell lines, nude mice	up-regulation	qRT-PCR	FBXW7	cell cycle arrest	**LRA**, antagomiR-25 TF *in vitro* and *in vivo*, WB	40 GC vs. ADJNTT	[129]
16	GC	human GC tissue and human BP (Chinese), human GC cell lines	up-regulation	qRT-PCR	LATS2	apoptosis growth inhibition	**DLRA**, antagomiR-25 and miR-25 TF	14 GC vs. 14 CONT	[131]
17	GC	human GC tissue (Chinese), human GC cell lines	up-regulation	qRT-PCR	RECK	metastasis and invasion inhibitor	**LRA**, antagomiR-25 and miR-25 TF, WB	27 GCs	[132]
18	GC	human GC tissue and human BP (Chinese)	up-regulation of the miR-106b-25 cluster	qRT-PCR	N/A	N/A	N/A	40 GC vs. ADJNTT	[130]
19	GC	human BP (Chinese)	up-regulation	Taqman low density array, qRT-PCR	N/A	N/A	N/A	160 CONT, 124 GNCA, 36 GCA	[135]
20	GC	human BP (Chinese)	up-regulation	Exiqon miRCURY ready to use PCR human panel- I+II-V.M for 168 miRNAs	N/A	N/A	N/A	133 GC vs. 109 CONT	[134]
21	GC	human GC tissue (EPIC-EURGAST study)	up-regulation of the miR-106b-25 cluster	Applied Biosystems Big Dye Terminators Cycle Sequencing Kit	N/A	N/A	N/A	365 GC vs. 1284 CONT	[124]
22	GC	human GC tissue (Korean)	up-regulation	qRT-PCR	N/A	N/A	N/A	91 GC vs. 26 CONT	[143]
23	CRC	human CC cell lines	up-regulation	ArrayExpress microRNA microarray chip, qRT-PCR	MCU	apoptosis	**LRA**, IF	N/A	[138]
24	CRC	human BS (Chinese)	up-regulation of the miR-106b-25 cluster	qRT-PCR	N/A	N/A	N/A	66 CRC vs. 86 CONT	[139]
25	CRC	human CRC tissue (Japanese)	up-regulation of the miR-106b-25 cluster	Agilent miRNA microarray, Early Access Version, qRT-PCR	N/A	N/A	N/A	13 CRC vs. 4 CONT	[141]
26	CRC	human CRC tissue (Chinese)	up-regulation	qRT-PCR	N/A	N/A	N/A	186 CRC vs. 18 CONT	[140]
27	CRC	human CRC tissue (Chinese), BALB/c-nu nude mice, human CRC cell line	*down-regulation*	qRT-PCR	SMAD7	proliferation, metastasis and invasion	**LRA**, miR-25 TF, WB	20 CRC vs. ADJNTT	[142]

Abbreviations: ADJNTT: adjacent non-tumorous tissue, BP: blood plasma, BS: blood serum, CAC: cholangiocarcinoma, CONT: control, CRC: colorectal cancer, DLRA: dual luciferase reporter assay, EAC/BE: esophageal adenocarcinoma/Barrett esophagus, ESCC: esophageal squamous cell carcinoma, GC: gastric cancer, GCA: gastric cardia adenocarcinoma, GNCA: gastric non-cardia adenocarcinoma, IF: immunofluorescence, IH: immunohistochemistry, LRA: luciferase reporter assay, KO: knock out, OE: overexpression, TF: transfection, TG: transgene, WB: Western blot. The abbreviations in bold are considered as gold standard methods for miRNA target validation.

#### Esophageal squamous cell carcinoma

Esophageal squamous cell carcinoma (ESCC) is the predominant histological subtype of esophageal carcinomas in Asian countries accounting for approximately 90% of all esophageal tumors [120]. The prognosis is very poor due to early invasion and metastasis via the well-developed network of submucosal lymphatic vessels [115]. Xu et al. reported that the up-regulation of miR-25 in ESCC samples of Chinese patients significantly correlated with the presence of lymph node metastases and advanced TNM stage [114]. In this study, miR-25 directly targeted E-cadherin (CDH1) and inhibited its metastasis suppressor function and promoted cell migration and invasion in human ESCC cell lines (KYSE-150 and KYSE-410) [114] (Table 7) (Figure 3). Fang et al. demonstrated that miR-25 could down-regulate desmocollin-2 (DSC2) in human ESCC cell lines (EC109, KYSE150, KYSE180 and SHEEC) [121] (Table 7) (Figure 3). The repression of DSC2 was proposed to increase the level of free γ-catenin. It competes with β-catenin to form a complex with E-cadherin [121]. This competition might subsequently cause an increase in free cytoplasmic β-catenin level leading to its enhanced nuclear transport and transcriptional activity. This activates invasion-associated gene expression and ESCC invasion [121]. Zhu et al. found in 5 North Chinese ESCC patients that miR-25 was significantly overexpressed in ESCC tissues as compared to normal basal or differentiated cells [122] (Table 7). A clinical study enrolling 194 ESCC patients and 94 healthy volunteers in China showed that miR-25 was overexpressed both in serum and tissue samples of ESCC patients [123] (Table 7). The overexpression of miR-25 was associated with lymph node metastasis [123] (Table 7). Another clinical study recruiting 111 Chinese ESCC patients confirmed that miR-25 was overexpressed in serum and tissue samples of ESCC patients [123] (Table 7). However, overexpression of miR-25 showed a positive correlation with overall survival in this study [123] (Table 7). Moreover, a third clinical study enrolling 105 Japanese ESCC patients and 50 healthy controls also reported that miR-25 level was significantly higher in plasma and tissues samples of ESCC patients [119] (Table 7). Plasma miR-25 levels were significantly reduced in postoperative samples as compared to those preoperatively, while increased again if ESCC recurred [119] (Table 7).

### Gastric cancer

Gastric cancer (GC) ranks the second among cancer-related mortality causes [124]. Over 95% of gastric tumors are adenocarcinomas [125]. Proximal and distal gastric adenocarcinomas are the two main clinical manifestations of GC [124]. Histologically, there are two major types: 1) intestinal type presenting clearly defined glandular structures (54% of cases) and 2) diffuse type adenocarcinoma consisting of individually infiltrating neoplastic cells (32% of cases) [124, 126]. In addition, there is an indeterminate type as an uncommon variant (15% of cases) [124, 126]. Interestingly, the highest incidence of GC is frequently found in Eastern Asia especially in China, Eastern Europe and South America, and the lowest in North America and most parts of Africa [126]. A number of studies found unequivocally that miR-25 is overexpressed in human GC cell lines, tissue and serum/plasma samples as well. A mechanistic study by Petrocca et al. found that E2F1 (also called retinoblastoma binding protein 3) induced the overexpression of the miR-106b/25 cluster via its host gene MCM7 [125] (Table 7). Moreover, miR-106b and miR-93 could control E2F1 by a negative feedback loop that might be important in preventing E2F1 self-activation and, possibly, apoptosis [125] (Table 7) (Figure 3). In addition, overexpression of the miR-106b/25 cluster resulted in a decreased response of gastric cancer cells to TGFβ interfering with the synthesis of cell cycle inhibitor p21 (also called CDKN1A) and the pro-apoptotic Bim (also called BCL2L11)[125] (Table 7) (Figure 3). Interestingly, another study using human gastric cell lines also reported that the amplification of MCM7 and its intron miR-25 might be the major molecular switchers in the development of gastric cancer [127]. This study reported that MCM7 and miR-25 were able to suppress the adjacent gene, collagen type I alpha 2 chain (Col1a2) as well as the tumor suppressor p53 and activate the proto-oncogene tyrosine-protein kinase c-Src gene in human GC cell lines [127]. Li et al. showed overexpression of miR-25 in plasma and tissue samples of GC patients which promoted gastric cancer migration, invasion and proliferation by directly targeting the tumor suppressor TOB1 (also known as transducer of ERBB2, 1 or transducer of Her1/2) and correlated with poor survival [126] (Table 7) (Figure 3). In contrast, a single nucleotide polymorphism rs41274221 in the mature miR-25 gene was associated with reduced tumor growth and metastasis in GC patients [128]. In this study, the overexpression of miR-25 with AA genotype resulted in the reduced ability of binding on the direct target TOB1 mRNA as compared with GG or GA phenotypes [128] (Table 7) (Figure 3). The reduced binding ability of mir-25 resulted in protective effects in gastric cancer cell lines in this study [128] (Table 7) (Figure 3). Others demonstrated that overexpression of miR-25 promoted cell proliferation, invasion and migration by directly down-regulating the tumor suppressor E3 ubiquitin ligase FBXW7 and up-regulating its substrates including G1/S-specific cyclin E1 (CCNE1) and v-myc avian myelocytomatosis viral oncogene homolog (MYC) in human GC samples and cell lines [129, 130] (Table 7) (Figure 3). It was also reported that miR-25 and miR-107 could simultaneously reduce the expression of large tumor suppressor 2 (LATS2) gene promoting cell growths and invasion in human gastric cancer cell lines [131] (Table 7) (Figure 3). Zhao et al. published that miR-25 may directly target the tumor suppressor reversion-inducing-cysteine-rich protein with kazal motifs (RECK) inducing cell growth and motility in human GC tissues and cell lines [132] (Table 7) (Figure 3). RECK has been reported to suppress matrix metalloproteinases including MMP-2, MMP-9, and MMP-14 which are also involved in angiogenesis and cancer progression related breakdown of the extracellular matrix [132]. Clinical studies have also found that miR-25 is elevated in tissue and/or plasma/serum samples of GC patients (stage I-III) from China [133–136], Korea [137] and Europe [124] (EPIC-EURGAST study).

### Colorectal cancer

Colorectal cancer is the second most common cancer among men and women worldwide. Mir-25 has been shown to directly target an important factor in inflammatory carcinogenesis, the angiopoietin-like protein 2 and the mitochondrial calcium uptake regulating mitochondrial calcium uniporter in cells and colorectal carcinoma cell lines (Table 7) (Figure 3) [138]. Clinical studies have also found that miR-25 is overexpressed in colorectal cancer tissue and serum samples of Chinese [139, 140] and Japanese patients [141] showing correlation with TNM stage and patient prognosis [140] (Table 7). In contrast, Li et al. found that miR-25 was downregulated and its direct target Smad7 – a TGF-β type 1 receptor superfamily member - was upregulated in human colorectal cancer tissue samples of Chinese patients [142] (Table 7) (Figure 4).

### Hepatocellular carcinoma

Hepatocellular carcinoma (HCC) is the third most common cause of cancer-related mortality with the highest incidence in Asia and Africa [143]. A number of studies have unequivocally shown overexpression of miR-25 and the miR-106b/25 cluster in HCC cell lines, clinical HCC tissue, and serum samples. Mir-25 has been reported to reduce apoptosis by directly targeting the pro-apoptotic Bim (also known as BCL2L11) in human liver cell lines and HCC tumor tissues [144]. MiR-25 has also reduced the expression of TNF-related apoptosis-inducing ligand (TRAIL) via the PTEN/PI3K/Akt/Bad axis in liver cancer stem cells [145], human cholangiocarcinoma cell lines, and tissue samples [146] (Table 8) (Figure 3). Bim has also been shown to be a direct target of miR-25 in ovarian cancer, esophageal adenocarcinoma, and gastric cancer. Overexpression of miR-25 and reduction of TRAIL-induced apoptosis has also been associated with thyroid cancer [147]. Interestingly, WNT/beta-catenin pathway was reported to induce miR-25 expression leading to the repression of its direct target RhoGDI1 and overexpression of the epithelial-mesenchymal transition inducing SNAIL in HCC cell lines and human HCC tissues [148] (Table 8) (Figure 3). Another study using hepatoma and normal hepatic cell lines demonstrated that miR-25 gene and other 4 miRNA genes were in hypomethylation status and these miRNAs were also upregulated in HCC [149] (Table 8) (Figure 3). Overexpression of the miR-106b-25 cluster and repression of its direct target retinoblastoma 1 (RB1) oncogene and histone acyltransferase (KAT2b) were also reported in regenerating liver after 2/3 partial hepatectomy [150] (Table 8) (Figure 3). This observation might have clinical translational value in the renewal of HCC because partial hepatectomy is a commonly performed operation to treat hepatic tumors. Another study reported that miR-25 directly repressed cytochrome P450 2B6. This member of the cytochrome P450 family is responsible for the metabolism of nearly 25% of drugs including several anticancer agents (e.g., tamoxifen, cyclophosphamide, and iphosphamide, *etc*.) [151] (Table 8) (Figure 3).

**Table 8:** The role of miR-25 in hepatocellular carcinoma

	Disease	Species and tissue or cell type	Alteration of miR-25 expression	Method for miR-25 detection	Target gene	Biological function	Method for target validation	Sample size in clinical studies	Ref.
1	HCC	human HCC tissues (Asian), human HCC cell cultures	up-regulation of the miR-106b-25 cluster	qRT-PCR	BIM, E2F1	apoptosis, cell cycle arrest	**LRA**, antagomiR-25 TF, WB	55 HCC vs. ADJNTT	[145]
2	HCC	liver cancer stem cells	up-regulation	qRT-PCR	PTEN	metastasis and invasion inhibitor	antagomiR-25 TF	N/A	[146]
3	HCC	human HCC tissue (Chinese), human HCC cell lines	up-regulation	qRT-PCR	RhoGDI1	metastasis and invasion inhibitor	**LRA**, WB, miRNA or siRNA TF, IF	35 HCC vs. ADJNTT	[148]
4	HCC	human HCC cell lines	up-regulation	MeDip chip	N/A	N/A	bioinformatics	N/A	[150]
5	HCC	human HCC tissue (Chinese)	up-regulation	qRT-PCR	N/A	N/A	N/A	133 HCC vs. ADJNTT	[153]
6	HCC(HBV, HCV)	human BS (Chinese)	up-regulation	Solexa sequencinq, RT-PCR	N/A	N/A	N/A	210 CONT, 135 HBV, 48 HCV, 120 HCC	[154]
7	HCC (HBV)	human BS (Chinese)	up-regulation	qRT-PCR	N/A	N/A	N/A	23 HBV pos. HCC, 20 liver cirrhosis, 20 chronic hepatitis B, 16 CONT	[149]
8	HCC (HBV)	human HBV pos. HCC tissue human BP (Chinese)	up-regulation	Taqman low density array, qRT-PCR	N/A	N/A	N/A	50 HCC vs. 37 cancer free HBV pos.	[156]
9	HCC (HBV)	human HBV pos. HCC tissue (Chinese), human HCC cell lines	up-regulation of the miR-106b-25 cluster and MCM7	qRT-PCR	N/A	N/A	N/A	120 HCC vs. ADJNTT	[157]
10	HCC	human HCC cells	up-regulation of the miR-106b-25 cluster	Northern blot	N/A	N/A	N/A	N/A	[144]
11	CAC	human CAC tissue (American), human CAC cell lines	up-regulation	qRT-PCR	TRAIL	apoptosis	**LRA**, IF	15 CAC vs. 4 CONT	[147]

Abbreviations: ADJNTT: adjacent non-tumorous tissue, BS: blood serum, CAC: cholangiocarcinoma, CONT: control, DLRA: dual luciferase reporter assay, HBV: hepatitis B virus, HCC: hepatocellular carcinoma, HCV: hepatitis C virus, IF: immunofluorescence, IH: immunohistochemistry, LRA: luciferase reporter assay, KO: knock out, OE: overexpression, TF: transfection, TG: transgene, WB: Western blot. The abbreviations in bold are considered as gold standard methods for miRNA target validation.

A clinical study enrolling 131 Chinese HCC patients has reported that overexpression of miR-25 in human HCC tissue showed a negative correlation with overall survival [152] (Table 8). Another study recruiting 513 subjects from China showed that elevation of miR-25 in blood serum could be a biomarker of HCC [153] (Table 8). Another cohort study enrolling Chinese patients with HBV-related small HCC (23 cases), liver cirrhosis (20 cases), chronic hepatitis B (20 cases) and healthy controls (16 cases) has found that serum miR-25 could be a potential biomarker not only of HBV-related HCC but also of liver cirrhosis and chronic hepatitis B infection [149] (Table 8). Interestingly, clinical studies reported that increased levels of circulating miR-25 were positively correlated with liver diseases in children with cystic fibrosis as well [154] (Table 8). Additional studies reported that the miR-106b-25 cluster or miR-25 alone was overexpressed in HBV positive HCC tissue and serum samples [149, 155, 156] (Table 8). Moreover, transfection of human HCC cells with hepatitis B virus X protein resulted in overexpression of the miR-106b-25 cluster and its host gene MCM7 [157] (Table 8).

## CONCLUSIONS

**Figure 5:**
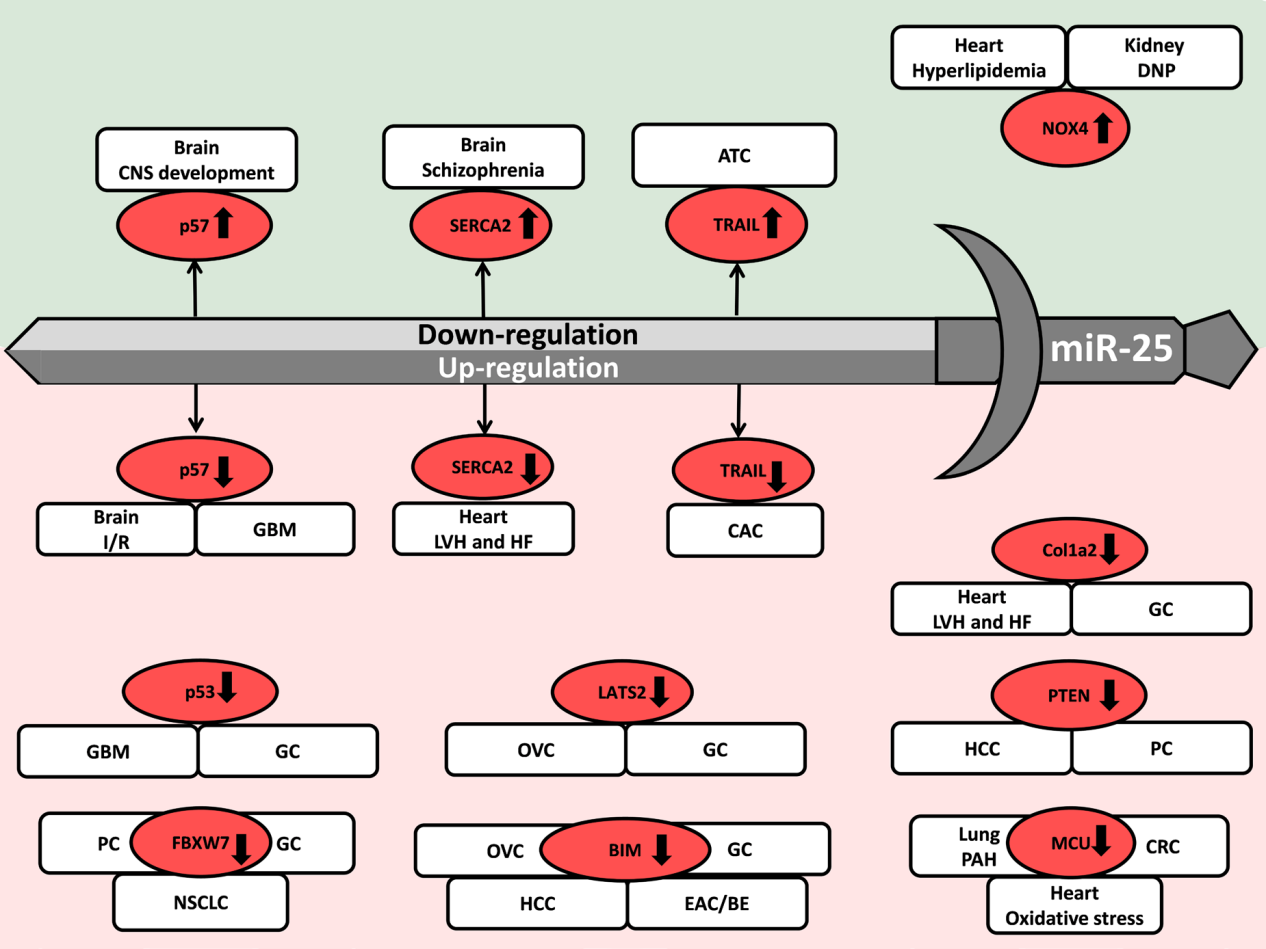
MiR-25 acts as a double-edged sword in the development of diverse diseases ATC: anaplastic thyroid cancer, CAC: cholangiocarcinoma, CNS: central nervous system, CRC: colorectal cancer, DNP: diabetic nephropathy, EAC/BE: esophageal adenocarcinoma/Barrett esophagus, GC: gastric cancer, GBM: glioblastoma multiforme, HCC: hepatocellular carcinoma, LVH: left ventricular hypertrophy, HF: heart failure, I/R: ischemia/reperfusion, NSCLC: non-small cell lung carcinoma, OVC: ovarian cancer, PAH: pulmonary arterial hypertension, PC: prostate cancer. Genes in red bubbles are targets of miR-25 in multiple organs/diseases.

In the past decade, a growing body of evidence showed that miRNAs are key regulators of different human diseases. These miRNAs could be potentially new therapeutic targets in the future. MiR-25 is expressed in a wide variety of tissues and cell types targeting many mRNAs. In this review, we have shown that both overexpression and repression of miR-25 could result in the development of different diseases (Figure 5). Expressional change of miR-25 seems to act as a double-edged sword in case of three target mRNAs including p57, SERCA2, and TRAIL. Overexpression or repression of these target mRNAs causes different diseases in the same (p57) or different tissues (SERCA2 and TRAIL) (Figure 5). Moreover, there are common target mRNAs of miR-25 in different tissue and cell types regulating the same biological process. The expressional changes of these common target mRNAs could result in different diseases sharing common general pathomechanisms (e.g., NOX4 regulating oxidative stress in heart and kidney diseases, Col1a2 in myocardial fibrosis and tumor metastasis, BIM in different cancer types, etc.) (Figure 5). Therefore, long-term miR-25 based therapy seems to be possible in the diseased target cells. Nevertheless, potentially harmful effects of miR-25 based drugs (antagomiR-25 or miR-25 mimic) could originate not only from off-target side effects (unwanted gene expression changes or chemical toxicity) but also could result from on-target side effects in non-targeted and non-diseased tissues.

On the other hand, different molecular mechanisms may lead to the development of a specific disease and these molecular mechanisms could be regulated by different miRNAs. Therefore, miRNAs could be diagnostic and prognostic biomarkers of certain diseases. MiR-25 seems to be an important biomarker in cancerous diseases, however, expressional level of miR-25 alone does not seem to be sufficient to set up diagnosis or assess disease progression. Expressional change of miR-25 among a specific set of miRNAs or other biomarkers might be more reliable in the diagnosis or prognosis of a specific disease, however, validation of the usefulness of such diagnostic panels still need to be done.
